# AIE Multinuclear Ir(III) Complexes for Biocompatible Organic Nanoparticles with Highly Enhanced Photodynamic Performance

**DOI:** 10.1002/advs.201802050

**Published:** 2019-01-21

**Authors:** Liping Zhang, Yuanyuan Li, Weilong Che, Dongxia Zhu, Guangfu Li, Zhigang Xie, Nan Song, Shi Liu, Ben Zhong Tang, Xingman Liu, Zhongmin Su, Martin R. Bryce

**Affiliations:** ^1^ Key Laboratory of Nanobiosensing and Nanobioanalysis at Universities of Jilin Province Department of Chemistry Northeast Normal University 5268 Renmin Street Changchun Jilin Province 130024 P. R. China; ^2^ State Key Laboratory of Polymer Physics and Chemistry Changchun Institute of Applied Chemistry Chinese Academy of Sciences Changchun 130022 P. R. China; ^3^ Department of Chemistry Hong Kong Branch of Chinese National Engineering Research Center for Tissue Restoration and Reconstruction Division of Life Science State Key Laboratory of Molecular Neuroscience Institute for Advanced Study Institute of Molecular Functional Materials The Hong Kong University of Science and Technology Clear Water Bay Kowloon Hong Kong 999077 China; ^4^ Department of Chemistry Durham University Durham DH1 3LE UK

**Keywords:** aggregation‐induced emission, in vivo, multinuclear Ir(III) complexes, nanoparticles, photodynamic therapy

## Abstract

The singlet oxygen (^1^O_2_) generation ability of a photosensitizer (PS) is pivotal for photodynamic therapy (PDT). Transition metal complexes are effective PSs, owing to their high ^1^O_2_ generation ability. However, non‐negligible cellular toxicity, poor biocompatibility, and easy aggregation in water limit their biomedical applications. In this work, a series of red‐emitting aggregation‐induced emission (AIE) Ir(III) complexes containing different numbers of Ir centers (mono‐, di‐, and trinuclear) and the corresponding nanoparticles (NPs) AIE‐NPs, are designed and synthesized. The increase of ^1^O_2_ generation ability is in line with the increasing number of Ir centers. Compared with the pure Ir(III) complexes, the corresponding NPs offer multiple advantages: (i) brighter emission; (ii) higher phosphorescence quantum yields; (iii) longer excited lifetime; (iv) higher ^1^O_2_ generation ability; (v) better biocompatibility; and (vi) superior cellular uptake. Both in vitro and in vivo experiments corroborate that AIE‐NPs with three iridium centers possess potent cytotoxicity toward cancer cells and effective inhibition of tumor growth. To the best of knowledge, this work is the first example of NPs of multinuclear AIE Ir(III) complexes as PSs for enhanced PDT. This study offers a new method to improve the efficiency of PSs for clinical cancer treatments.

## 1 Introduction

Photodynamic therapy (PDT) has attracted great attention in recent years due to the noninvasive nature, high specificity, controllability, and insignificant side effects compared with traditional surgery, chemotherapy, and radiotherapy.^1^ The key element for PDT is that highly cytotoxic reactive oxygen species (ROS) generated by the photosensitizers (PSs) upon irradiation lead to cell apoptosis and necrosis.^2^ An excellent PS is crucial for the therapeutic efficacy of PDT. At present, most PSs for PDT are based on organic dyes, including boron dipyrromethene (BODIPY), porphyrin, and their derivatives.^3^ However, the reported PSs possess several drawbacks: (i) the relatively low molar extinction coefficient results in high dosages and longer irradiation time;^4^ (ii) the poor water solubility and stability severely hinder their application in biological conditions;^5^ (iii) undesirable aggregation‐caused quenching (ACQ) of emission in aqueous media reduces ROS generation.^6^ Therefore, it is highly desirable to design PSs that can circumvent these shortcomings.

An effective method for improving the efficiency of singlet oxygen generation is to accelerate the intersystem crossing (ISC) by introducing heavy atoms into PSs, such as halogens, transition metals, etc.^7^ Especially, Ir(III) complexes have attracted considerable attention as PSs because of their ideal photophysical properties, large Stokes shift, and high ISC ability.^8^ For example, Huang and co‐workers designed a mitochondria‐targeted Ir(III) complex as a PS to improve PDT effects under hypoxia.^9^ An ingenious organoiridium PS that can induce specific oxidative attack on proteins within cancer cells was constructed by Sadler and co‐workers.^10^ The previous reports mainly focused on mononuclear Ir(III) complexes, whereas research into multinuclear Ir(III) complexes as PSs has been overlooked. We envisaged that additional metal centers will increase metal‐to‐ligand charge transfer (^3^MLCT), which will further result in an increase of molar absorption coefficient, and improved generation of ^1^O_2_.^11^ Multinuclear Ir(III) complexes could, therefore, be highly efficient PSs for PDT.

Ir(III) complexes possess non‐negligible cellular toxicity and water insolubility, resulting in poor biocompatibility and limited biomedical applications.^12^ Recently, the strategy of polymer‐encapsulated nanoparticles (NPs) has successfully solved these problems.^13^ Unfortunately, the NP formulations are not suitable for traditional PSs owing to ACQ of emission.[[qv: 7a]] In 2001, Tang and co‐workers discovered aggregation‐induced emission (AIE),^18^ by which fluorescence is intensified in an aggregate state through restriction of intramolecular motions (RIM) which prohibits the dissipation of energy.^15^ Since then, AIE materials have been exploited in optoelectronic and biological applications. Our group has reported dinuclear AIE Ir(III) complexes using Schiff bases as both chelate and bridging motifs.^16^ The flexibility of the diimine spacer permits the ligands to rotate and bend freely, and they can adopt the optimum coordination geometries of the metal ions. Furthermore, triphenylamine (TPA), with a propeller‐like structure and strong electron donating ability, has been used to construct red‐emitting AIE molecules.^17^ Red emission is desirable as it can reduce interference from the background autofluorescence and increase the depth of penetration in tissue.^18^ Is it possible to combine the advantages of multinuclear transition metal complexes and NPs simultaneously to enhance the PDT effect? Inspired by this idea, we synthesized a series of red‐emitting AIE Ir(III) complexes and their corresponding NPs and explored their potential as PSs for clinical cancer treatments.

## 2 Results and Discussion

**Figure 1:**
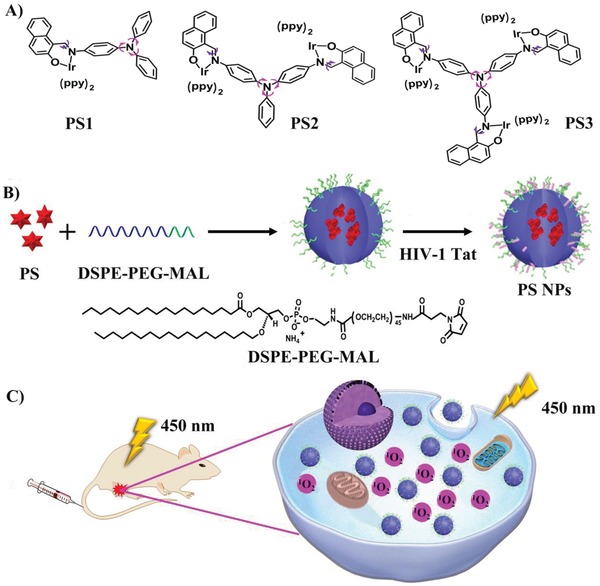
A) Chemical structures of **PS1**, **PS2**, and **PS3**; B) The synthesis of NPs; C) Schematic illustration of **PS3** NPs as PSs for PDT.

In this work, TPA is used as a bridge to obtain Schiff ligands which can electronically couple one to three metal centers. A series of red‐emitting AIE Ir(III) complexes were obtained: mononuclear, dinuclear, and trinuclear complexes (**Figure** **1**A), named **PS1**, **PS2**, and **PS3**, respectively, with **PS1** serving as a model for the multinuclear analogs. The corresponding NPs, named **PS1** NPs, **PS2** NPs, and **PS3** NPs, were obtained by polymer‐encapsulation methods (Figure 1B).^19^ A schematic illustration of how **PS3** NPs were successfully used for PDT is shown in Figure 1C. The results establish that the NPs possess highly effective ^1^O_2_ generation ability, good biocompatibility, negligible dark toxicity, superior cell uptake, enhanced PDT activity, and tumor inhibition.

The synthetic routes to **PS1**, **PS2**, and **PS3** are shown in Scheme S1 (Supporting Information). Their chemical structures and purity were validated by proton and carbon nuclear magnetic resonance (^1^H and ^13^C NMR) spectroscopy (Figures S1–S12 and Figures S13–S15, Supporting Information), mass spectrometry (Figures S16–S21, Supporting Information), and elemental analysis. The X‐ray molecular structure of **PS1** is shown in Figure S23 (Supporting Information). The highest occupied molecular orbital (HOMO) and lowest unoccupied molecular orbital (LUMO) distributions of **PS1**, **PS2**, and **PS3** are shown in Figure S22 (Supporting Information). Subsequently, the NPs were obtained by using the iridium complexes as the core, biocompatible 1,2‐distearoyl‐*sn*‐glycero‐3‐phosphoethanolamine‐*N*‐[maleimide(poly(ethyleneglycol))‐2000] (DSPE‐PEG‐MAL) as the encapsulation matrix, and the HIV‐1 transactivator (RKKRRQRRRC) as the surface functionalization group (Figure 1B). HIV‐1 Tat is the cell penetrating peptide, which can effectively transport NPs into cells.

**Figure 2:**
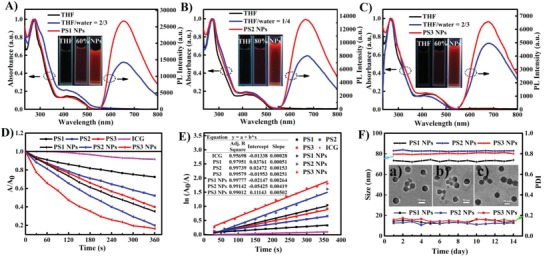
A) UV–vis absorption spectra and emission spectra of **PS1** in THF, THF/water (v:v) = 2/3, and **PS1** NPs in water (λ_ex_ = 469 nm), inset: emission images of the **PS1** and **PS1** NPs under 365 nm UV illumination; B) UV–vis absorption spectra and emission spectra of **PS2** in THF, THF/water (v:v) = 1/4, and **PS2** NPs in water (λ_ex_ = 469 nm), inset: emission images of the **PS2** and **PS2** NPs under 365 nm UV illumination; C) UV–vis absorption spectra and emission spectra of **PS3** in THF, THF/water (v:v) = 2/3, and **PS3** NPs in water (λ_ex_ = 469 nm), inset: emission images of the **PS3** and **PS3** NPs under 365 nm UV illumination; D) Comparison of the decay rates of different PSs under irradiation (450 nm, 20 mW cm^−2^), *A*_0_ = absorption of ICG without irradiation. *A* = real‐time absorption of ICG with different irradiation time; E) Time‐dependent ^1^O_2_ generation kinetics. *A*_0_ = absorption of ICG without irradiation. *A* = real‐time absorption of ICG with different irradiation time; F) Stability of size distribution of changes of different PSs during 14 d, inset: the TEM images of (a) **PS1** NPs, (b) **PS2** NPs, and (c) **PS3** NPs. (**PS1** or **PS2** or **PS3** or **PS1** NPs or **PS2** NPs or **PS3** NPs) = 10^−5^ m, (ICG) = 6.5 × 10^−6^ m.

The UV–vis absorption and photoluminescence (PL) spectra of **PS1**, **PS2**, and **PS3** and the corresponding NPs are shown in **Figure** **2**A–C, respectively. The intense absorption bands at around 250–350 nm are attributed to spin‐allowed (π–π*) transitions of the ligands. The weak absorption bands from 370 to 520 nm are assigned to metal‐to‐ligand charge transfer (^3^MLCT) and ligand‐to‐ligand charge transfer (^1^LLCT) features.^20^ The molar absorption coefficient at 450 nm increases with the number of metal centers for the Ir(III) complexes and their NPs in the order: **PS1** (*ɛ* = 7660 m ^−1^ cm^−1^) < **PS2** (*ɛ* = 16 176 m ^−1^ cm^−1^) < **PS1** NPs (*ɛ* = 17 570 m ^−1^ cm^−1^) < **PS3** (*ɛ* = 31 143 m ^−1^ cm^−1^) < **PS2** NPs (*ɛ* = 43 651 m ^−1^ cm^−1^) < **PS3** NPs (*ɛ* = 72 935 m ^−1^ cm^−1^) (Figure S25, Supporting Information). The absorbance of **PS1** NPs and **PS3** are 2.29 and 4.07 times higher than **PS1**, respectively. **PS1**, **PS2**, and **PS3** are almost nonemissive in pure tetrahydrofuran (THF) solution (Figure S26, Supporting Information). However, PL intensities of **PS1**, **PS2**, and **PS3** were significantly enhanced when the water fraction of water‐THF mixtures reached 60%, 80%, and 60%, respectively, revealing an obvious AIE effect. Compared with the Ir(III) complexes, the corresponding NPs exhibit similar emission peaks but with brighter emission in water. The PL maxima of **PS1**, **PS2**, and **PS3** are 652, 671, and 690 nm, respectively, demonstrating a sequential redshift with the increasing number of metal centers. The large Stokes shifts and red emission of these PSs can improve the signal/background ratios by reducing interference from the background.[[qv: 17a]] The photoluminescence quantum yields (PLQYs) of **PS1**, **PS2**, and **PS3** NPs (33%, 15%, and 35%, respectively) in water are higher than those of the corresponding Ir(III) complexes (11%, 5%, and 8%, respectively) in THF‐water mixtures (Table S1, Supporting Information). The excited‐state lifetimes (τ) of **PS1** NPs, **PS2** NPs, and **PS3** NPs are 4.92, 5.38, and 4.61 µs in water, while those of **PS1**, **PS2**, and **PS3** are 959.17, 90.32, and 75.98 ns in THF‐water mixtures, respectively (Table S1 and Figure S27A,B, Supporting Information). These results confirmed the successful encapsulation of the Ir(III) complexes into the polymer matrix.

High ^1^O_2_ generation ability is imperative for effective PDT.^21^ The ^1^O_2_ generation ability of **PS1**, **PS2**, and **PS3** and the corresponding NPs were evaluated by measuring the absorbance of indocyanine green (ICG) upon light irradiation.[[qv: 3f,4]] A continuous decrease of absorbance at 790 nm (the specific absorption of ICG) was observed upon irradiation of ICG (6.5 nmol) solutions containing **PS1**, **PS2**, and **PS3** and the corresponding NPs (10 nmol), respectively (Figure 2D; Figure S31, Supporting Information). This decrease in absorbance of ICG is limited due to the low power of the irradiation (20 mW cm^−2^) and the illumination wavelength (450 nm) which is outside the absorption range of ICG.[[qv: 3f,7a]] On the contrary, negligible attenuation in absorbance was found in three control groups: (i) ICG with irradiation; (ii) **PS1**, **PS2**, and **PS3** and the NPs, with irradiation, respectively; (iii) ICG solutions containing **PS1**, **PS2**, and **PS3** and the NPs without irradiation, respectively (Figures S28–S30, Supporting Information). These results suggest that the PSs generate ^1^O_2_ upon irradiation and show excellent photostability. As shown in Figure 2E, ^1^O_2_ generation of **PS1**, **PS2**, and **PS3** and the corresponding NPs conform to first‐order kinetics. The slope follows the order: **PS1** (0.00051) < **PS2** (0.00153) < **PS3** (0.00251) < **PS1** NPs (0.00264) < **PS2** NPs (0.00419) < **PS3** NPs (0.00502). A steeper slope indicates a greater ability to generate ^1^O_2_. It is worth mentioning that the slopes of **PS1** NPs and **PS3** NPs are 5.17 and 4.92 times higher than for **PS1** and **PS3**, respectively. As anticipated, the increase of slope is in keeping with the number of metal centers in the PSs, and the slopes for the NPs are much higher than for the Ir(III) complexes. The data suggest that within this series **PS3** NPs should be the most effective for PDT. These results are clearly distinct from a recent report of monomeric, dimeric, and trimeric BODIPY derivatives and their derived NPs.[[qv: 3g]] In the BODIPY series increased π‐conjugated coupling between the BODIPY units induced a redshift in absorption, and produced dually cooperative phototherapy. The BODIPY NPs showed no significant influence on ^1^O_2_ generation compared to the isolated BODIPY dyes. This is in contrast to the Ir complexes and their NPs in the present study, where ^3^MLCT transitions play an important role at the metal centers and ^1^O_2_ generation is improved.

The morphology, size, and stability of the NPs were compared.[[qv: 5c,18]] Transmission electron microscopy (TEM) images show that the **PS1** NPs, **PS2** NPs, and **PS3** NPs exhibit spherical morphology and uniform dispersion in water with average diameters of 44, 47, and 45 nm, respectively (Figure 2F, inset). Meanwhile, dynamic light scattering (DLS) demonstrated that the average sizes of **PS1** NPs, **PS2** NPs, and **PS3** NPs are 73, 83, and 79 nm, respectively (Table S2 and Figure S32, Supporting Information). The sizes obtained by DLS are larger than those obtained by TEM because a hydrated layer forms on the NPs in the aqueous system.[[qv: 5c]] Spherical NPs with dimensions of less than 100 nm are more easily endocytosed by cells. In addition, the size and size distributions of **PS1** NPs, **PS2** NPs, and **PS3** NPs collected in water for 14 d by DLS (Figure 2F) were almost unchanged. Such high stability is beneficial to circulation of particles in blood.[[qv: 5a]] Consequently, the **PS1** NPs, **PS2** NPs, and **PS3** NPs possess spherical morphology, appropriate size, and high stability. These properties will facilitate their ensuing application in living cells and animals.

**Figure 3:**
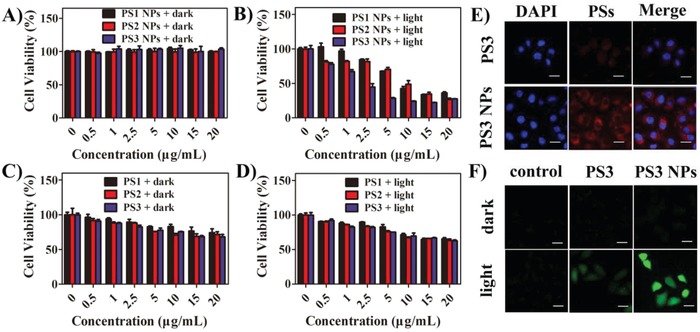
Cell viability of different PSs against HeLa cells A,C) under dark and B,D) under light (450 nm, 20 mW cm^−2^, 30 min); E) CLSM images of HeLa cells incubated with **PS3** and **PS3** NPs (20 µg mL^−1^) for 6 h, the scale bars are 20 µm; F) Generation of intracellular ROS mediated by **PS3** and **PS3** NPs upon irradiation (450 nm, 20 mW cm^−2^, 20 min) as indicated by the fluorescence of DCF.

In order to quantitatively evaluate the PDT effect, the cytotoxicity of **PS1**, **PS2**, and **PS3** and the corresponding NPs against HeLa cells were measured by 3‐(4,5‐dimethylthiazol‐2‐yl)‐2,5‐diphenyltetrazolium bromide (MTT) assays. After incubation of the HeLa cells with **PS1** NPs, **PS2** NPs, and **PS3** NPs (0–20 µg mL^−1^) for 24 h, the cell viability was still higher than 95% (**Figure** **3**A), indicating the good cytocompatibility and negligible dark cytotoxicity. However, under light irradiation (20 mW cm^−2^) for 30 min, the cell viability was obviously reduced (Figure 3B), implying potent phototoxicity. The half‐maximal inhibitory concentration (IC_50_) follows the order: **PS1** NPs (1.1 × 10^−5^ m) > **PS2** NPs (6.3 × 10^−6^ m) > **PS3** NPs (1.1 × 10^−6^ m). A smaller IC_50_ indicates an improved effect for PDT. The PDT effects of Ir(III) complexes **PS1**, **PS2**, and **PS3** were evaluated. As shown in Figure 3C,D, they showed some dark cytotoxicity but only slight phototoxicity. These results clearly demonstrate that polymer‐encapsulation is an efficient way to address biocompatibility issues, and the PDT effect can be improved by increasing the number of metal centers in PSs.

The advantages of effective ^1^O_2_ generation ability and low half‐maximal inhibitory concentration motivated us to further investigate intracellular behavior of **PS3** NPs. The cellular uptake of **PS3** and **PS3** NPs was investigated in HeLa by using confocal laser scanning microscopy (CLSM). The cell nuclei were stained with 4,6‐diamidino‐2‐phenylindole (DAPI). As shown in Figure 3E, red fluorescence was observed from cells treated with **PS3** NPs, which is significantly stronger than that with **PS3**. The enhanced cellular uptake is highly advantageous to improve PDT. The intracellular ^1^O_2_ generation was also investigated by using 2′,7′‐dichlorofluorescein diacetate (DCFH‐DA) as an indicator (Figure 3F). Before light irradiation, negligible green fluorescence was observed from all the cells, indicating an absence of ^1^O_2_ generation. Conversely, green emission was observed from both **PS3** and **PS3** NPs in the presence of irradiation. As expected, the green emission from **PS3** NPs is obviously stronger than that from **PS3**, which suggests that the ^1^O_2_ generation ability of NPs is higher than that of the pure Ir(III) complexes. In addition, the PDT effects of **PS3** and **PS3** NPs were also confirmed by the live/dead staining experiments (Figure S34, Supporting Information). After irradiation, almost all of the cells were killed by **PS3** NPs, and the efficiency of inducing cell death was higher than that of **PS3**. This result is in accord with the results of the MTT assay.

**Figure 4:**
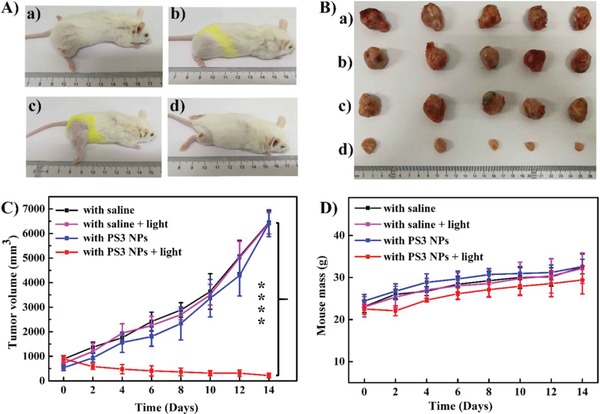
A) Representative images of mice. The hair on the thigh was removed immediately before irradiation. The images were taken on day 14 after irradiation and the different hair length on the different mice is due to an inconsistent rate of hair growth. B) Harvested tumors from various groups treated (a) with saline, (b) with saline and light, (c) with **PS3** NPs, (d) with **PS3** NPs and light (100 mg mL^−1^, 100 µL), light irradiation (450 nm, 200 mW cm^−2^, 20 min). C) Tumor volume measurement for different groups of mice (****, *P* < 0.0001, *n* = 5 per group, PDT vs other groups). D) Body weights of mice for different groups of mice.

Encouraged by the good performance of **PS3** NPs in cellular experiments, their potential for tumor inhibition was investigated in vivo. The murine models were established by subcutaneously injecting murine H22 cells into the right thigh. To explore the optimal time for light irradiation of **PS3** NPs in vivo, IR780 was loaded in **PS3** NPs. As shown in Figure S37 (Supporting Information), the fluorescence intensity of NPs in the tumor gradually increased within 12 h and then decreased, which indicated that 12 h after injection is the optimal time for light irradiation. The tumor‐bearing mice were then randomly divided into four groups and were intravenously injected with saline (groups 1), with saline and light (450 nm, 200 mW cm^−2^, 20 min) (group 2), with **PS3** NPs (100 µg mL^−1^, 100 µL) (group 3), and with **PS3** NPs and light (group 4). The tumor volume and body weight of the mice were measured every 2 d for two weeks. As shown in **Figure** **4**A–C, in the groups 1, 2, and 3, the relative tumor volumes showed a 8–9 times increase after 14 d, suggesting that only irradiation, or only **PS3** NPs, has no influence on tumor growth. In contrast, the significantly reduced tumor volume in the group 4 mice indicates the good antitumor performance of **PS3** NPs under light irradiation, which is significantly different from the control groups. Furthermore, the systemic toxicity of various treatments was evaluated via the mice's body weight changes and the histological slices. In comparison with group 1, negligible body weight losses were observed in groups 2, 3, and 4 (Figure 4D). The mice were sacrificed at day 14, and major organs and the tumors were collected for hematoxylin and eosin (H&E) staining (Figure S36, Supporting Information). The **PS3** NPs lead to destructive cell necrosis in the tumor under irradiation, indicating a severe cell injury. No pathological changes were found in the liver, heart, spleen, kidney, and lung in the four groups, indicating that **PS3** NPs are not significantly toxic in vivo. These results reveal that **PS3** NPs are suitable for in vivo PDT applications.

## 3 Conclusions

In summary, we have established for the first time that the efficiency of PDT can be enhanced by increasing the number of metal centers in PSs. Three red‐emitting AIE Ir(III) complexes with a different number of metal centers (mono‐, di‐, and trinuclear) and their corresponding NPs, were rationally designed and synthesized. The molar absorption coefficient increases with the number of metal centers, resulting in significantly improved ^1^O_2_ generation ability of the PSs. The nanoparticle formulation also further enhances the absorbance and ^1^O_2_ generation. In particular, the trinuclear **PS3** NPs are excellent for PDT due to the following properties: λ_max_^em^ at ≈690 nm, high phosphorescence quantum yields (35%), long excited state lifetime (4.61 µs), high molar absorption coefficient (*ɛ* = 72 935 m ^−1^ cm^−1^), excellent ^1^O_2_ generation ability, and negligible dark toxicity. Moreover, the **PS3** NPs upon irradiation can efficiently inhibit tumor growth in mice after tail vein injection. This study provides new insights into the design of highly efficient PSs for PDT in clinical therapeutics. Future work will include the development of multinuclear Ir(III) complexes with long excitation wavelength for in vivo imaging and PDT.

## Conflict of Interest

The authors declare no conflict of interest.

## Supporting information

Supplementary
