# Supplementary material for: AIE Multinuclear Ir(III) Complexes for Biocompatible Organic Nanoparticles with Highly Enhanced Photodynamic Performance
Source: Adv Sci (Weinh). 2019 Jan 21;6(5):1802050. doi: 10.1002/advs.201802050 (PMC6402395; doi:10.1002/advs.201802050)
Supplement: Supplementary file 1 — Supplementary [file ADVS-6-1802050-s001.pdf]

Copyright WILEY-VCH Verlag GmbH & Co. KGaA, 69469 Weinheim, Germany, 2019.

ADVANCED SCIENCE Open Access

Supporting Information

for *Adv. Sci.,* DOI: 10.1002/advs.201802050

AIE Multinuclear Ir(III) Complexes for Biocompatible Organic Nanoparticles with Highly Enhanced Photodynamic Performance

Liping Zhang, Yuanyuan Li, Weilong Che, Dongxia Zhu, Guangfu Li, Zhigang Xie,* Nan Song, Shi Liu, Ben Zhong Tang,* Xingman Liu, Zhongmin Su,* and Martin R. Bryce**

Copyright WILEY-VCH Verlag GmbH & Co. KGaA, 69469 Weinheim, Germany, 2019.

Supporting Information

AIE Multinuclear Ir(III) Complexes for Biocompatible Organic Nanoparticles with Highly Enhanced Photodynamic Performance

Liping Zhang, Yuanyuan Li, Weilong Che, Dongxia Zhu,* Guangfu Li, Zhigang Xie,* Nan Song, Shi Liu, Ben Zhong Tang,* Zhongmin Su,* Xingman Liu and Martin R. Bryce*

Experimental Section

Materials and Instrumentation：Materials obtained from commercial suppliers were used without further purification unless otherwise stated. All glassware, syringes, magnetic stirring bars and needles were thoroughly dried in a convection oven. All other materials for organic synthesis were purchased from Energy Chemical Company. 1,2-Distearoyl-sn-glycero-3-phosphoethanolamine-N-[maleimide(polyethylene glycol)-2000](DSPE-PEG-Mal) was provided by Shanghai Ponsure Biotech, Inc. HIV-1 transactivator of transcription (Tat) protein-derived cell penetrating peptide (C-terminus with cysteine) was purchased from GL Biochem Co., Ltd. (Shanghai, China). Indocyanine green (ICG) was purchased from Fisher Scientific. Dulbecco's Modified Eagle Medium (DMEM), RPMI-1640 Medium, and fetal bovine serum (FBS) were purchased from Sigma-Aldrich. DAPI was purchased from Life Technologies. LiveDead Cell Staining Kit was purchased from KeyGen Biotech Co., Ltd.. 2’,7’-Dichlorofluorescence diacetate (DCFH-DA) were purchased from Shanghai Beyotime Biotechnology Co., Ltd..

Reactions were monitored using thin layer chromatography (TLC). Commercial TLC plates were used and the spots were visualized under UV light at 254 and 365 nm. ^{1}H NMR spectra were recorded at 25 ºC on a Varian 500 MHz spectrometer. The chemical shifts (δ) are given in parts per million relative to internal standard TMS. The ^{1}H NMR spectra were referenced internally to the residual proton resonance in DMSO-d_6 (δ 2.49 ppm) or $CDCl_3$ (δ 7.24 ppm). UV-vis absorption spectra were recorded on a Shimadzu UV-3100 spectrophotometer. The photoluminescence spectra, excited state lifetimes (τ) and photoluminescence quantum yields (Φ_{PL}) were recorded on an Edinburgh FLS920 spectrofluorimeter under air at room temperature. The X-ray crystal structure data of **PS1** was collected on a Bruker Smart Apex II CCD diffractometer with graphite-monochromated Mo Kα radiation (λ = 0.71069 Å) at room temperature. Crystallographic data (excluding structure factors) for the structure reported in this paper have been deposited with the Cambridge

Crystallographic Data Centre as supplementary publication no. CCDC-1866977. Copies of the data can be obtained free of charge from www.ccdc.cam.ac.uk/conts/retrieving.html

Molecular orbital distributions were calculated at the B3LYP/6-31G*/lanl2dz level using the Gaussian 09 program package.[1] The 6-31G* basis set was employed for H, C, N, O atoms, while the iridium atom was described by the Hay-Wadt effective core potential (ECP) and a double-ξ basis set LANL2DZ. Transmission electron microscopy (TEM) and electron diffraction analyses of the samples were obtained using a TECNAI F20 microscope. The samples were prepared by placing microdrops of the solution on a holey carbon copper grid. Diameter and diameter distribution of the nanoparticles were determined by a Malvern Zeta-sizer Nano instrument for dynamic light scattering (DLS). Confocal laser scanning microscopy (CLSM) images were taken using a Zeiss LSM 700 (Zurich, Switzerland).

Synthesis of **A1**: Triphenylamine (5.00 g, 20 mmol) and $Cu(NO_3)_2$ (3.23 g, 13 mmol) in acetic acid (60 ml) was stirred at room temperature for 12 h. The mixture was poured into water (500 mL), and then filtered. The crude product was purified by column chromatography with petroleum ether / ethyl acetate (100:1 v/v) as eluent. An orange-red solid was obtained (82% yield). ^1H NMR (600 MHz, $CDCl_3$, δ [ppm]): 8.02 (d, J = 6.0 Hz, 2H, ArH), 7.36 (t, J = 6.0 Hz, 4H, ArH), 7.22-7.17 (m, 6H, ArH), 6.92 (d, J = 9.5 Hz, 2H, ArH).

Synthesis of **A2**: Aniline (3.27 g, 40 mmol), 4-fluoronitrobenzene (11.7 g, 83 mmol) and potassium carbonate (20.0 g, 0.289 mol) in DMSO (60 ml) was stirred at 190 ^oC for 12 h. After cooling to room temperature, the mixture was poured into water (500 ml), and then filtered. After recrystallization from acetic acid a yellow solid was obtained (57% yield). ^1H NMR (500 MHz, $CDCl_3$, δ [ppm]): 8.17 (d, J = 8.5 Hz, 4H, ArH), 7.44 (t, J = 5.0 Hz, 2H, ArH), 7.32 (t, J = 5.0 Hz, 1H, ArH), 7.18-7.15 (m, 6H, ArH).

Synthesis of **A3**: 4-Nitroaniline (3.45 g, 25 mmol), 4-fluoronitrobenzene (7.88 g, 50 mmol) and potassium carbonate (20.0 g, 0.289 mol) in DMSO (60 ml) was stirred at 160 ^oC for 6 h. After cooling to room temperature, the mixture was poured into water (500 ml), and then

filtered. The solid was washed with hot methanol and nitrobenzene. A yellow solid was obtained (68%). ^{1}H NMR (500 MHz, DMSO-d_6, δ [ppm]): 8.26 (d, J = 7.5 Hz, 6H, ArH), 7.36 d, J = 7.5 Hz, 6H, ArH).

Synthesis of **B1**: A 100-mL two-necked flask, equipped with a reflux condenser and a dropping funnel, was charged with **A1** (5.00 g, 17 mmol), 10% Pd/C catalyst (0.862 g) and ethanol (50 ml). The mixture was stirred at 0 ^{o}C under a nitrogen atmosphere. Then, hydrazine monohydrate (10 ml) was slowly added dropwise and subsequently refluxed for 6 h. The mixture was filtered using a Buchner funnel and washed with THF. The filtrate was evaporated in a rotary evaporator, followed by recrystallization from ethanol to yield white crystals (92%). ^{1}H NMR (500 MHz, $CDCl_3$, δ [ppm]): 7.19 (t, J = 6.0 Hz, 4H, ArH), 7.02 (d, J = 6.0 Hz, 4H, ArH), 6.95 (d, J = 7.5 Hz, 2H, ArH), 6.91 (t, J = 6.5 Hz, 2H, ArH), 6.63 (d, J = 7.0 Hz, 2H, ArH), 3.59 (s, 2H, NH_2).

Synthesis of **B2**: The procedure was the same as that of **B1**, except **A2** was used instead of **A1**. White crystals were obtained (89% yield). ^{1}H NMR (500 MHz, $CDCl_3$, δ [ppm]): 7.12 (d, J = 6.5 Hz, 2H, ArH), 6.93 (d, J = 7.0 Hz, 4H, ArH), 6.88 (d, J = 6.5 Hz, 2H, ArH), 6.79 (t, J = 6.0 Hz, 1H, ArH), 6.60 (d, J = 7.0 Hz, 4H, ArH), 3.51 (s, 4H, NH_2).

Synthesis of **B3**: The procedure was the same as that of **B1**, except **A3** was used instead of **A1**. Gray crystals were obtained (83% yield). ^{1}H NMR (500 MHz, DMSO-d_6, δ [ppm]): 6.62 (d, J = 7.0 Hz, 6H, ArH), 6.51 (d, J = 7.5 Hz, 6H, ArH), 4.78 (s, 6H, NH_2).

Synthesis of **L1**: **B1** (0.260 g, 1.0 mmol) and 2-hydroxy-1-naphthaldehyde (0.206 g, 1.2 mmol) in ethanol (30 ml) were stirred at 79 ^{o}C for 8 h. After cooling to room temperature the precipitate was filtered and recrystallized from ethanol. An orange-red solid was obtained (87% yield). ^{1}H NMR (500 MHz, $CDCl_3$, δ [ppm]): 15.66 (s, 1H, OH), 9.32 (s, 1H), 8.09 (d, J = 7.0 Hz, 1H, CH), 7.78 (d, J = 7.5 Hz, 1H, ArH), 7.71 (d, J = 6.5 Hz, 1H, ArH), 7.51 (t, J = 6.0 Hz, 1H, ArH), 7.33 (t, J= 5.5 Hz, 1H, ArH), 7.29-7.25 (m, 6H, ArH), 7.15-7.04 (m, 9H, ArH). MS: (ESI-TOF) [m/z]: 413.5 (calcd: 414.4).

Synthesis of **L2**: **B2** (0.275 g, 1.0 mmol) and 2-hydroxy-1-naphthaldehyde (0.413 g, 2.4 mmol) in ethanol (30 ml) were stirred at 79 °C for 8 h. After cooling to room temperature the precipitate was filtered and recrystallized from ethanol. An orange-red solid was obtained (91% yield). ^{1}H NMR (500 MHz, $CDCl_3$, δ [ppm]): 15.62 (s, 2H, OH), 9.35 (s, 2H, CH), 8.11 (d, J = 7.0 Hz, 2H, ArH), 7.80 (d, J = 7.5 Hz, 2H, ArH), 7.72 (d, J = 6.5 Hz, 2H, ArH), 7.52 (t, J = 6.0 Hz, 2H, ArH), 7.35-7.30 (m, 8H, ArH), 7.20-7.12 (m, 6H, ArH), 7.10 (t, J =7.5 Hz, 3H, ArH). MS: (ESI-TOF) [m/z]: 582.6 (calcd: 583.2).

Synthesis of **L3**: **B3** (0.290 g, 1.0 mmol) and 2-hydroxy-1-naphthaldehyde (0.516 g, 3.6 mmol) in ethanol (30 ml) was stirred at 79 °C for 8 h. After cooling to room temperature the precipitate was then filtered and recrystallized from ethanol. An orange-red solid was obtained (93% yield). ^{1}H NMR (500 MHz, $CDCl_3$, δ [ppm]): 15.58 (s, 3H, OH), 9.39 (s, 3H, CH), 8.13 (d, J = 7.5 Hz, 3H, ArH), 7.81 (d, J = 7.5 Hz,3H, ArH), 7.73 (d, J = 7.5 Hz, 3H, ArH), 7.53 (t, J = 7.0 Hz, 3H, ArH), 7.35 (d, J = 7.0 Hz, 9H, ArH), 7.28 (t, J =8.5 Hz, 3H, ArH), 7.24 (d, J = 6.0 Hz, 3H, ArH), 7.12 (d, J = 8.0 Hz, 3H, ArH). MS: (ESI-TOF) [m/z]: 753.4 (calcd: 752.3).

Synthesis of **PS1**: A yellow suspension of the dichloro-bridged diiridium complex $[Ir(ppy)_2Cl]_2$ (0.107 g, 0.1 mmol),[2] **L1** (0.083 g, 0.2 mmol) and Na_2CO_3 (0.349 g, 3.2 mmol) in 2-ethoxyethanol (60 ml) was stirred at 120 °C for 8 h under a nitrogen atmosphere. After cooling to room temperature, the solution was extracted with ethyl acetate (150 ml) and washed with water (100 ml). The filtrate was evaporated in a rotary evaporator, the residue was purified by column chromatography with petroleum ether / dichloromethane (3:1 v/v) as eluent. An orange-red solid was obtained (81% yield). ^{1}H NMR (500 MHz, $CDCl_3$, δ [ppm]): 8.93 (d, J = 4.5 Hz, 1H, ArH), 8.89 (d, J =5.0 Hz 1H, ArH), 8.84 (s, 1H, CH), 7.83 (d, J = 7.0 Hz, 1H, ArH), 7.78 (d, J = 7.0 Hz, 1H, ArH), 7.64-7.60 (m, 3H, ArH),7.56 (t, J = 6.0 Hz, 3H, ArH), 7.34 (t, J = 9.5 Hz, 1H, ArH), 7.30 (d, J = 6.0 Hz, 1H, ArH), 7.21 (t, J = 7.0 Hz, 4H, ArH), 7.12 (t, J = 5.0 Hz, 1H, ArH), 7.07 (t, J = 5.0 Hz, 1H, ArH), 6.98-6.91 (m, 7H, ArH), 6.84 (t, J = 7.5 Hz, 2H, ArH), 6.75-6.69 (m, 2H, ArH), 6.56 (t, J = 6.5 Hz, 1H, ArH), 6.47 (d,

J =5.0 Hz, 2H, ArH), 6.34 (d, J = 6.5 Hz 1H, ArH), 6.21 (d, J = 6.5 Hz 1H, ArH), 6.08 (d, J = 7.0 Hz, 2H, ArH). ^{13}C NMR (151 MHz, $CDCl_3$, δ[ppm]): 168.63, 168.39, 167.30, 155.34, 152.65, 150.30, 149.28, 148.64, 147.92, 147.60, 144.61, 144.09, 144.03, 136.59, 135.36, 134.14, 133.08, 132.59, 129.21, 129.02, 128.93, 128.58, 127.89, 126.86, 126.62, 123.87, 123.70, 123.62, 123.43, 123.31, 122.21, 121.50, 121.18, 119.73, 118.94, 118.80, 117.78, 111.50. MS: (MALDI-TOF) [m/z]: 914.3 (calcd: 914.26). Anal. Calcd. for $C_{51}H_{37}IrN_4O$: C 67.0, H 4.08, N 6.13. Found C 67.1, H 4.07, N 6.12%. Crystals were obtained by diffusion of petroleum ether into a dichloromethane solution of **PS1**.

Synthesis of **PS2**: A yellow suspension of the dichloro-bridged diiridium complex $[Ir(ppy)_2Cl]_2$ (0.214 g, 0.2 mmol), **L2** (0.106 g, 0.2 mmol) and Na_2CO_3 (0.349 g, 3.2 mmol) in 2-ethoxyethanol (60 ml) were stirred at 120 ^oC for 8 h under a nitrogen atmosphere. After cooling to room temperature, the solution was extracted with ethyl acetate (150 ml) and washed with water (100 ml). The filtrate was evaporated in a rotary evaporator, the residue was purified by column chromatography with petroleum ether / dichloromethane/ acetone (40:60:1 v/v/v) as eluent. An orange-red solid was obtained (73% yield). ^{1}H NMR (600 MHz, $CDCl_3$, δ [ppm]): 8.93 (d, J = 4.2 Hz, 2H, ArH), 8.90 (d, J = 4.8 Hz, 2H, ArH), 8.85 (s, 2H, CH), 7.79-7.76 (m, 4H, ArH), 7.64-7.61 (m, 2H, ArH), 7.59-7.54 (m, 10H, ArH), 7.38-7.34 (m, 2H, ArH), 7.24-7.21 (m, 4H, ArH), 7.14-7.10 (m, 2H, ArH), 7.09-7.06 (m, 2H, ArH), 6.97-6.93 (m, 1H, ArH), 6.91 (t, J = 7.2 Hz, 2H, ArH), 6.87-6.71 (m, 6H, ArH), 6.74 (t, J = 7.2 Hz, 2H, ArH), 6.67-6.63 (m, 2H, ArH), 6.58-6.52 (m, 2H, ArH), 6.32 (t, J = 9.0 Hz, 6H, ArH), 6.24-6.20 (m, 2H, ArH), 6.05 (t, J =8.4 Hz, 4H, ArH). ^{13}C NMR (151 MHz, $CDCl_3$, δ[ppm]): 168.66, 168.37, 167.31, 155.31, 152.61, 150.50, 149.25, 148.26, 147.98, 147.52, 144.60, 144.08, 143.97, 136.63, 136.52, 135.34, 134.18, 133.08, 132.57, 129.23, 128.95, 128.73, 128.62, 127.89, 126.86, 126.64, 123.73, 123.57, 123.03, 122.98, 121.49, 121.21, 119.58, 118.91, 118.84, 117.71, 111.49. MS: (MALDI-TOF) [m/z]: 1583.4 (calcd: 1583.4). Anal.Calcd. for $C_{84}H_{59}Ir_2N_7O_2$: C 63.74, H 3.76, N 6.19. Found C 63.72, H 3.79, N 6.17%.

Synthesis of **PS3**: A yellow suspension of the dichloro-bridged diiridium complex $[Ir(ppy)_2Cl]_2$ (0.321 g, 0.3 mmol), **L3** (0.150 g, 0.2 mmol) and Na_2CO_3 (0.349 g, 3.2 mmol) in 2-ethoxyethanol (60 ml) was stirred at 120 ^{o}C for 8 h under a nitrogen atmosphere. After cooling to room temperature, the solution was extracted with ethyl acetate (150 ml) and washed with water (100 ml). The filtrate was evaporated in a rotary evaporator, the residue was purified by column chromatography with petroleum ether / dichloromethane / acetone (20:30:1 v/v/v) as eluent. An orange-red solid was obtained (65% yield). ^{1}H NMR (500 MHz, $CDCl_3$, δ [ppm]): 8.95 (d, J = 4.5 Hz, 3H, ArH), 8.92 (d, J = 4.5 Hz, 3H, ArH), 8.84 (s, 3H, CH, ArH), 7.84 (d, J = 6.5 Hz, 3H, ArH), 7.77 (d, J = 7.0 Hz, 3H, ArH), 7.76-7.51 (m, 18H, ArH), 7.37 (t, J = 5.5 Hz, 3H, ArH), 7.19-7.09 (m, 9H, ArH), 6.97-6.94 (m, 3H, ArH), 6.86 (t, J = 5.0 Hz, 6H, ArH), 6.75 (t, J = 6.0 Hz, 3H, ArH), 6.62-6.55 (m, 6H, ArH), 6.30-6.16 (m, 12H, ArH), 6.04 (t, J = 8.5Hz, 6H, ArH). ^{13}C NMR (151 MHz, $CDCl_3$, δ[ppm]): 168.51, 168.29, 167.01, 155.26, 152.71, 152.47, 150.38, 149.20, 147.94, 144.65, 144.05, 143.56, 136.71, 136.47, 135.27, 134.27, 132.97, 132.49, 129.15, 128.85, 128.57, 127.70, 126.86, 126.63, 123.76, 123.51, 122.71, 122.35, 121.49, 121.16, 119.52, 118.88, 117.74, 111.51. MS: (MALDI-TOF) [m/z]: 2252.5 (calcd: 2251.54). Anal.Calcd. for $C_{117}H_{81}Ir_3N_{10}O_3$: C 62.41, H 3.63, N 6.22. Found C 62.39, H 3.61, N 6.24%.

Synthesis of **PS1/PS2/PS3** NPs: A mixture of the iridium complex **PS1**, **PS2** or **PS3** (1 mg) and DSPE-PEG-MAL (2 mg) in THF (1 ml) was sonicated for 1 min, the mixture was poured into water (10 mL) and then was sonicated for 5 min. The solution was stirred at room temperature for 24 h to evaporate THF. Subsequently, HIV-1 Tat (500 μg/mL, 150 μL) was added into the above solution and stirred at room temperature for 12 h. The excess HIV-1 Tat was removed by dialysis using a 10 000 molecular weight cutoff membrane. NPs were obtained by filtration through a 0.2 μm syringe-driven filter.

Cell culture: HeLa cells (the human cervical cancer cell line) and H22 cells (the murine hepatic carcinoma cell line) were purchased from Jilin University and grown in Dulbecco's

modified Eagle's medium (DMEM, GIBCO) supplemented with 10% heat-inactivated fetal bovine serum (FBS, GIBCO), 100 U mL penicillin and 100 μg mL streptomycin (Sigma). The cells were maintained in a humidified incubator at 37 °C with 5% CO_2.

Cytotoxicity assay: HeLa cells were harvested in a logarithmic growth phase and seeded in 96-well plates at a density of 10^4 cells per well for 24 h. Then different concentrations of **PS1/PS2/PS3** and **PS1/PS2/PS3** NPs of various concentrations (0-20 μg mL^{-1}) were added into cell culture medium separately. After incubating for 6 h, the light group received irradiation (450 nm, 20 mW cm^{-2}) for 30 min, then continued incubation to 24 h. The dark control group received nothing and were incubated for the same time as the control group. Subsequently the MTT (20 μL) was added to the medium for 4 h. The absorbance of MTT was measured by a Bio-Rad 680 microplate reader at 490 nm.

Cell uptake by confocal laser scanning microscopy (CLSM): HeLa cells were seeded in 6-well culture plates at a density of 5×10^4 cells per well for 24 h. Then the **PS3** and **PS3** NPs (20 μg mL^{-1}) were added into the cell culture medium separately. They were incubated for 0.5 h, 2 h and 6 h. Subsequently, 4′,6-diamidino-2-phenylindole (DAPI) was used to stain the cell nuclei. Finally, the outcomes were observed by CLSM with excitation at 488 nm.

Intracellular ROS assays: HeLa cells were seeded in 6-well culture plates at a density of 5×10^4 cells per well for 24 h. Then the **PS3** and **PS3** NPs (10 μg mL^{-1}) were added into the cell culture medium separately. After incubating for 6 h, the light group received irradiation (450 nm, 20 mW cm^{-2}) for 20 min, whereas the dark control group received nothing. Then using the DMEM solution without FBS the cells were washed. The DMEM solution containing DCFH-DA (10^{-5} mol L^{-1}) was added and incubated for 30 min. The cells were observed as soon as possible by CLSM with excitation at 488 nm.

Live/ dead cell staining assays: HeLa cells were harvested in a logarithmic growth phase and seeded in 96-well plates at a density of 104 cells per well for 24 h. The **PS3** and **PS3** NPs (10 μg mL^{-1}) were added into cell culture medium separately. After incubating for 6 h, the

light group received irradiation (450 nm, 20 mW cm^{-2}) for 30 min, then incubation was continued to 24 h. The dark control group received nothing and was incubated for the same time as the control. After staining with calcein-AM/PI for 40 min, phosphate-buffered saline (PBS) was used to wash the cells. Finally the cells were observed by Nikon C1 Si laser scanning confocal microscopy.

Tumor-bearing mouse model: All animal studies were performed in strict accordance with the NIH guidelines for the care and use of laboratory animals (NIH Publication No. 85-23 Rev. 1985) and were approved by the guidelines of the Committee on Animal Use and Care of the Chinese Academy of Sciences. Male mice were purchased from Jilin University, China (6 weeks, 15–20 g). Inoculating the subcutaneous murine H22 cells (100 μL) into the right thigh established the tumor-bearing mouse model.

In vivo PDT: After one week, the mice were intravenously injected with **PS3** NPs (100 μg mL^{-1}, 100 μL), the control group received saline instead. After 12 h the tumor site of the mouse was subjected to laser irradiation (200 mW cm^{-2}) for 20 min. The tumor volume and body weight of the mice were measured every two days for 14 days. Histological analysis: The mice were sacrificed at day 14, and major organs and the tumors were collected and fixed in 4% paraformaldehyde. Then they were embedded into paraffin, and sliced at a thickness of 5 μm. Slices were stained with hematoxylin and eosin (H&E) and imaged by optical microscopy.

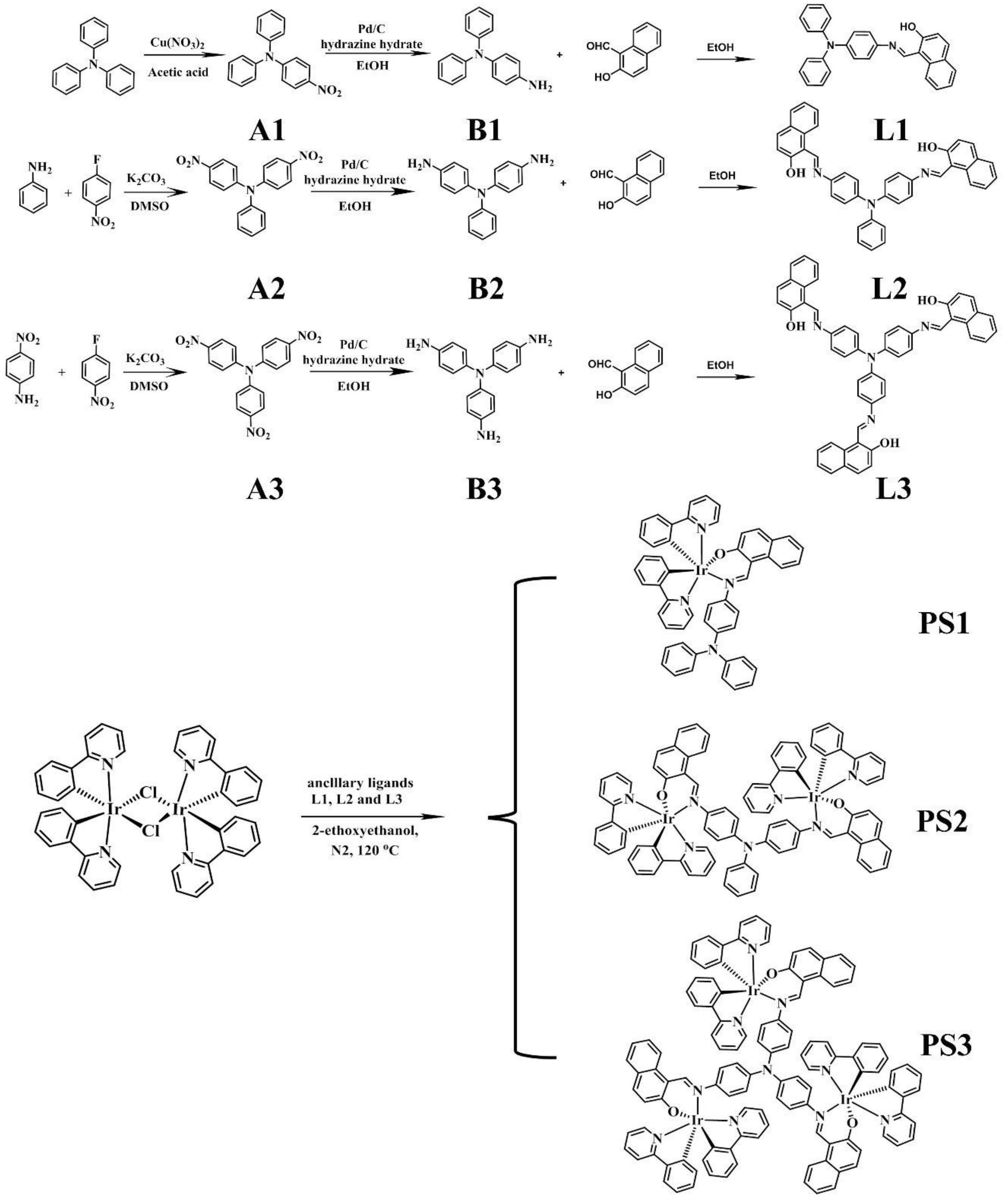

Scheme S1. Synthetic routes of **PS1**, **PS2** and **PS3**.

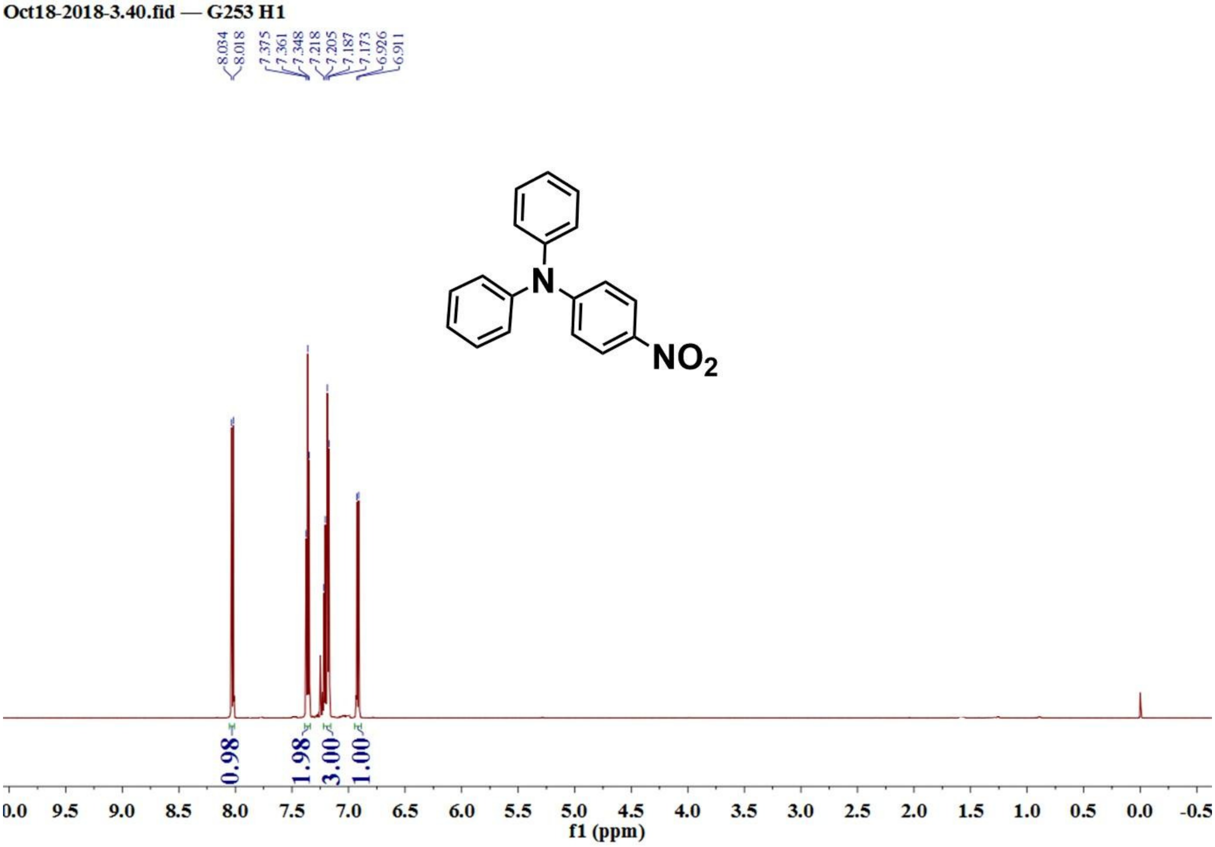

Figure S1. ^{1}H NMR spectrum of **A1** in $CDCl_3$.

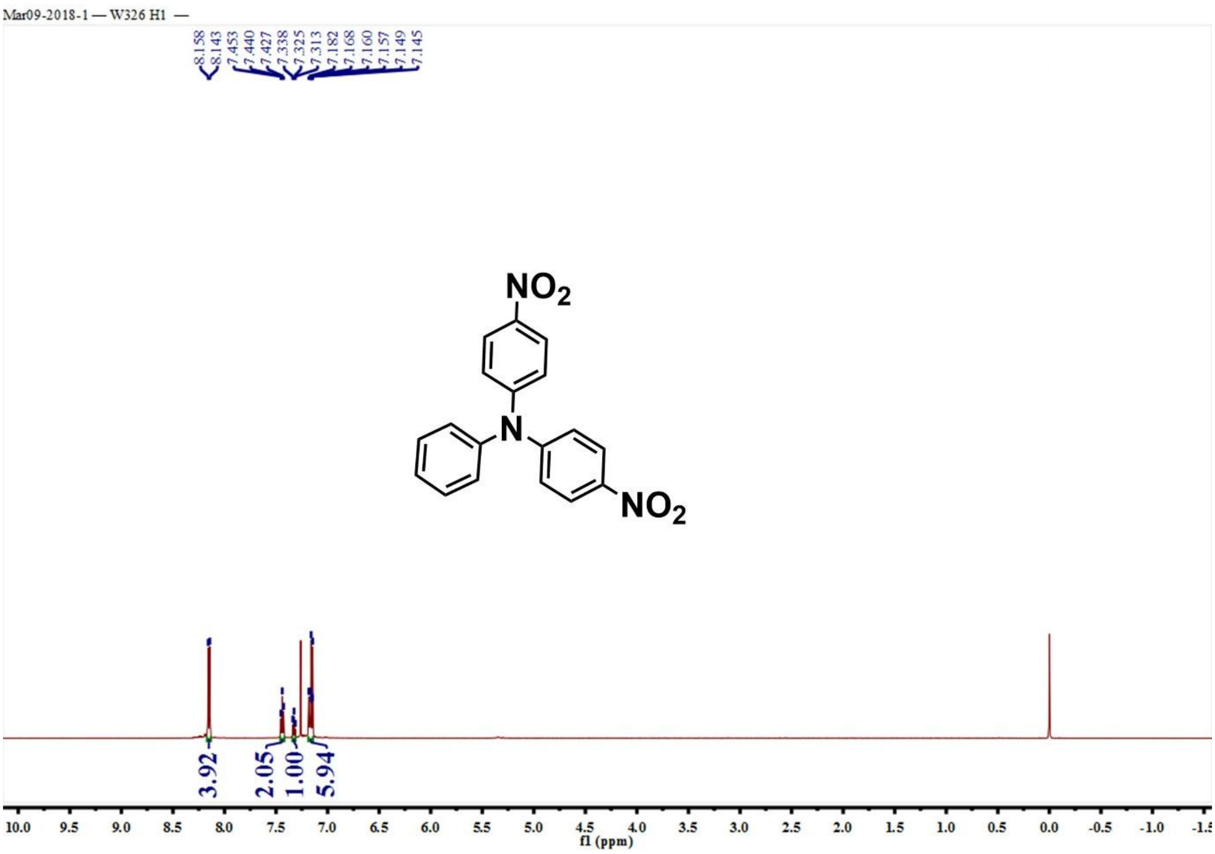

Figure S2. ^1H NMR spectrum of **A2** in $CDCl_3$.

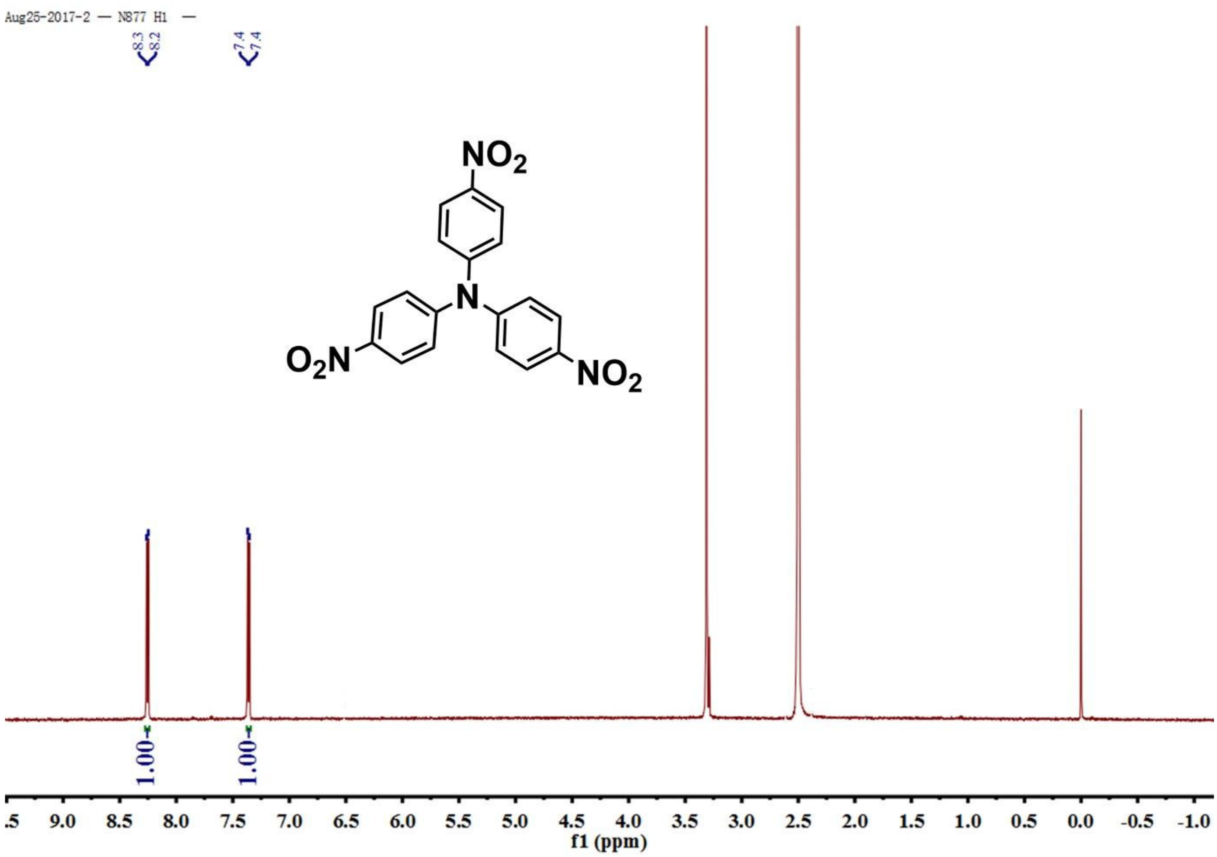

Figure S3. ^{1}H NMR spectrum of **A3** in DMSO-d_6.

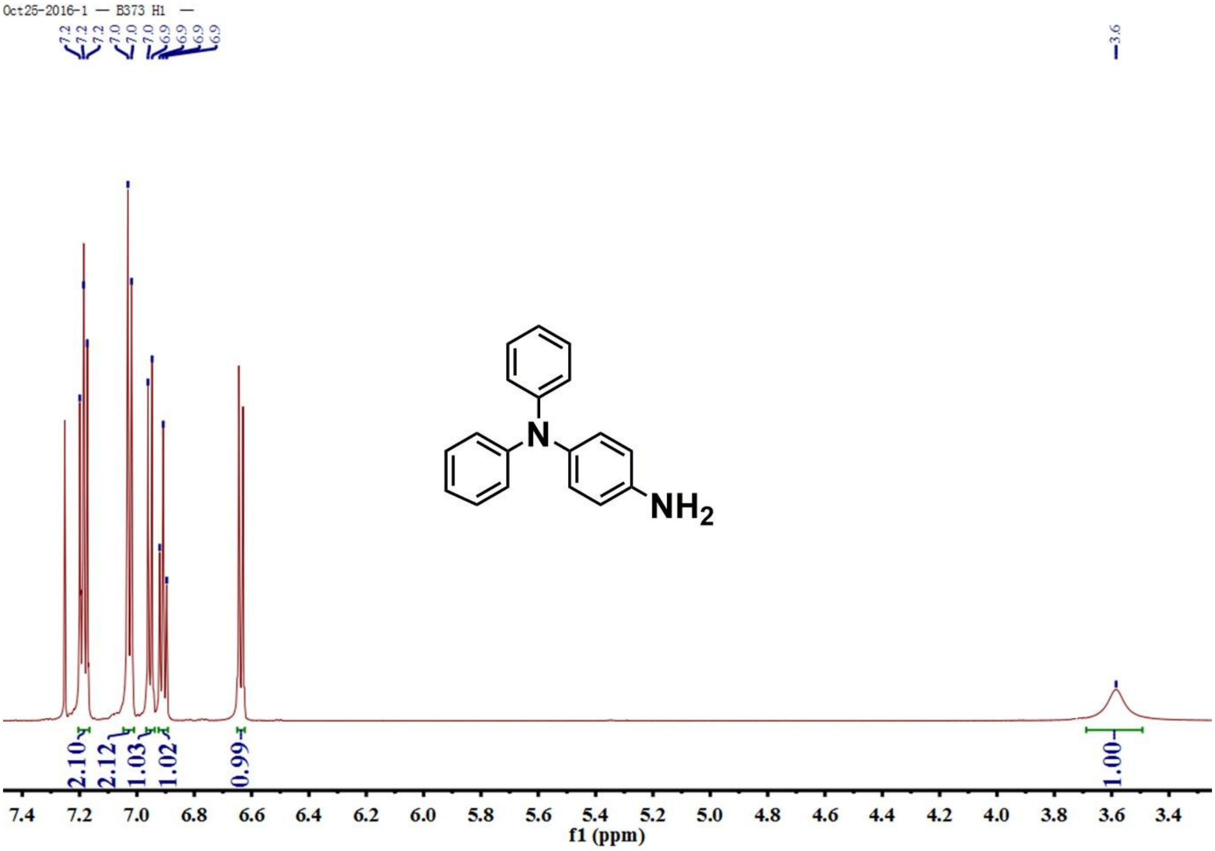

Figure S4. ^{1}H NMR spectrum of **B1** in $CDCl_3$.

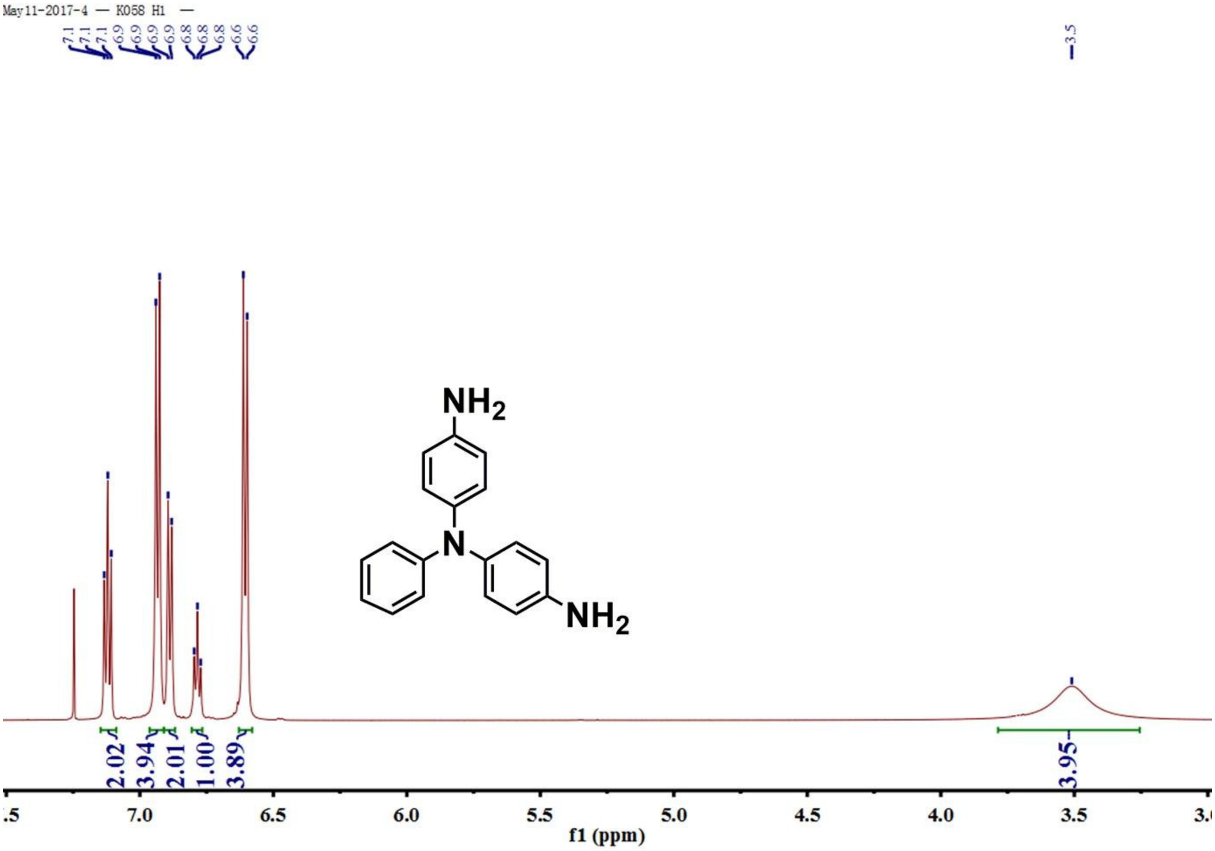

Figure S5. ^{1}H NMR spectrum of **B2** in $CDCl_3$.

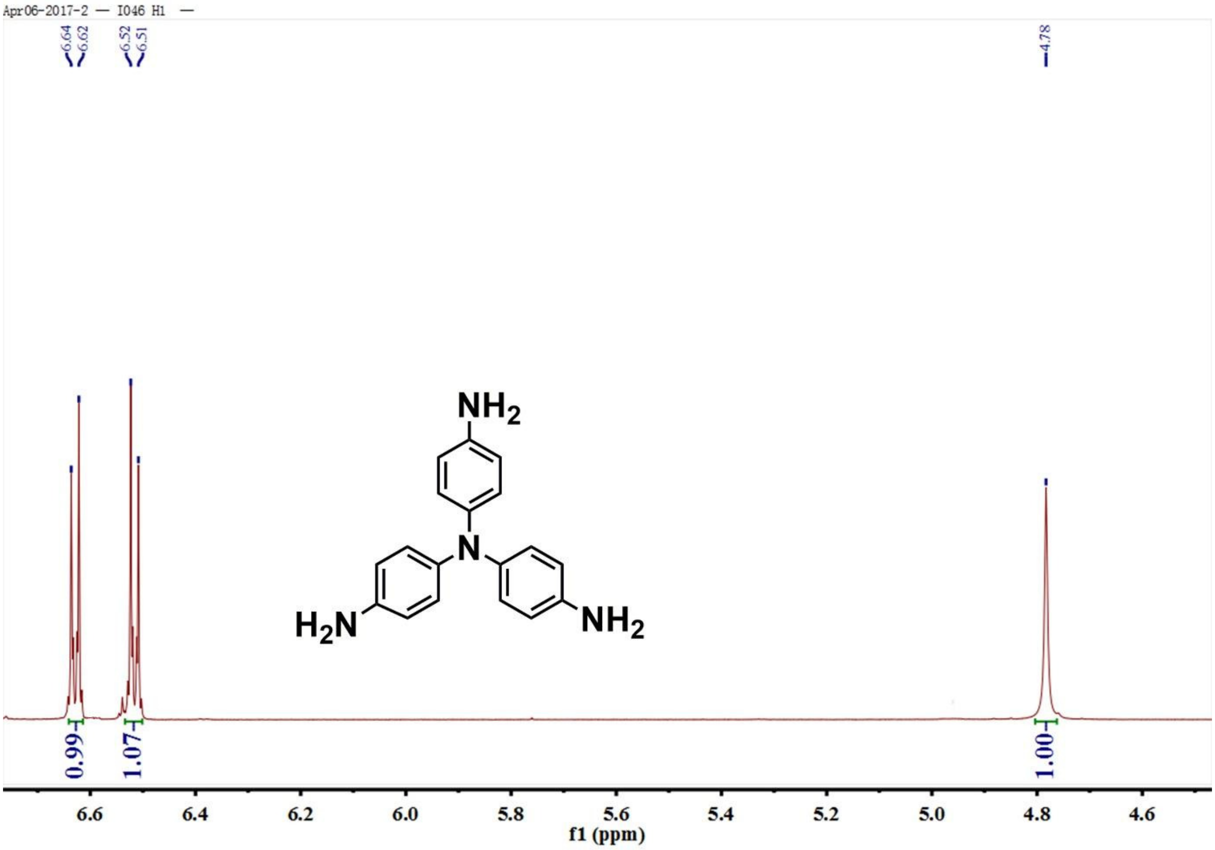

Figure S6. ^{1}H NMR spectrum of **B3** in DMSO-d$_6$.

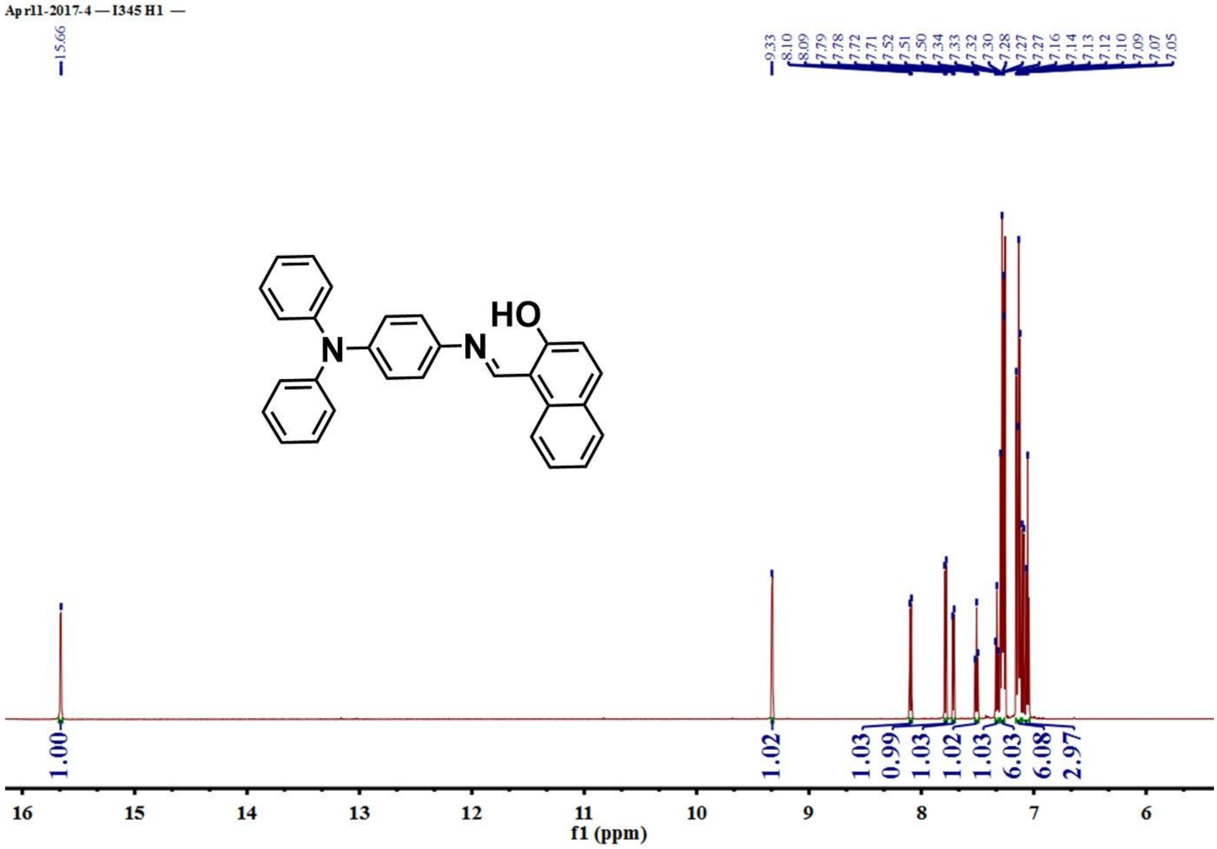

Figure S7. ^{1}H NMR spectrum of **L1** in $CDCl_3$.

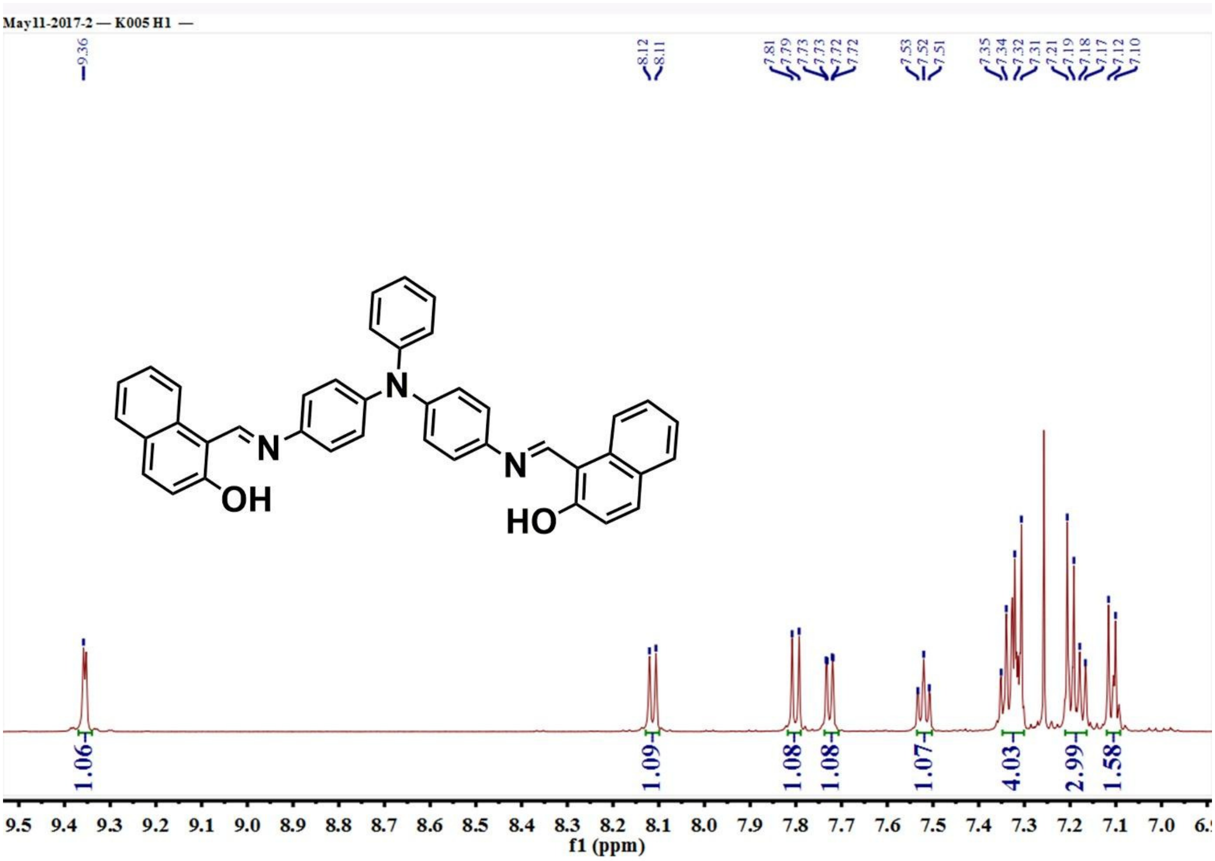

Figure S8. ^{1}H NMR spectrum of **L2** in $CDCl_3$.

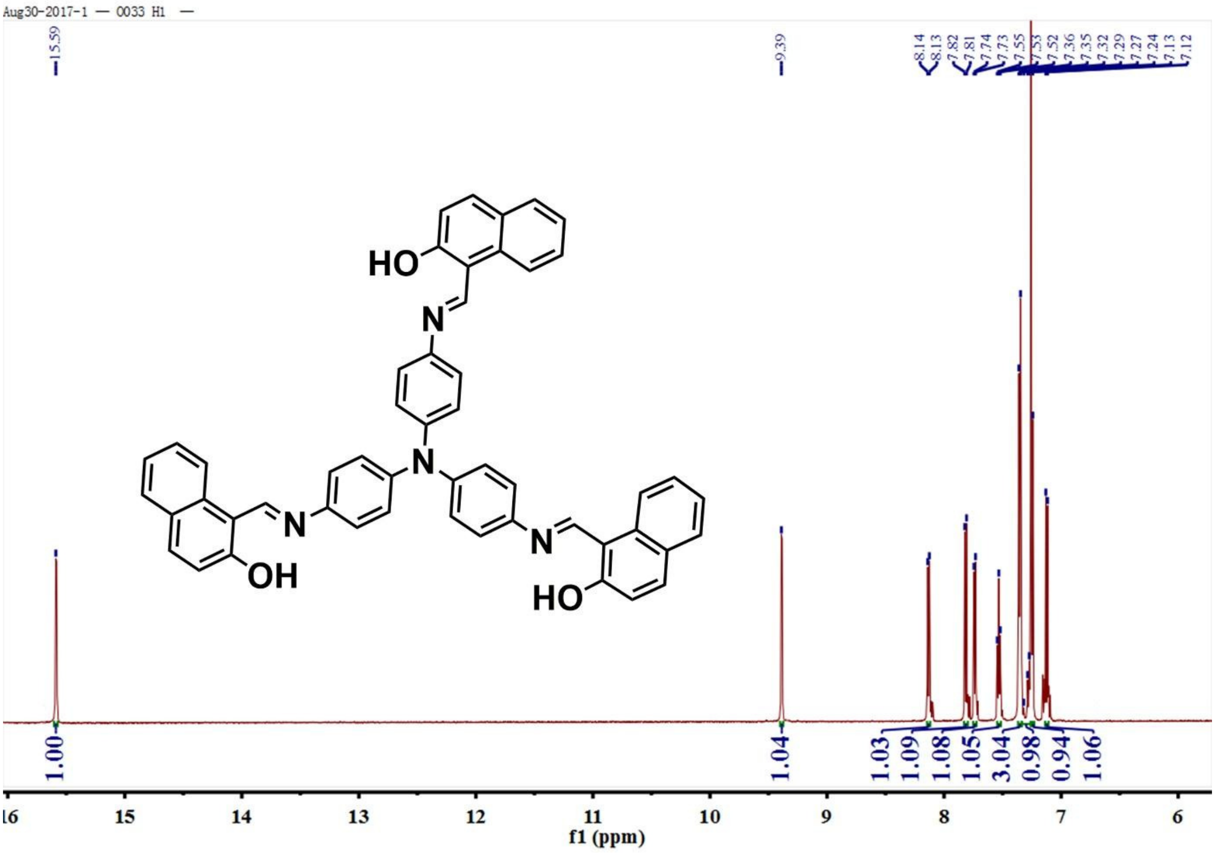

Figure S9. ^{1}H NMR spectrum of **L3** in $CDCl_3$.

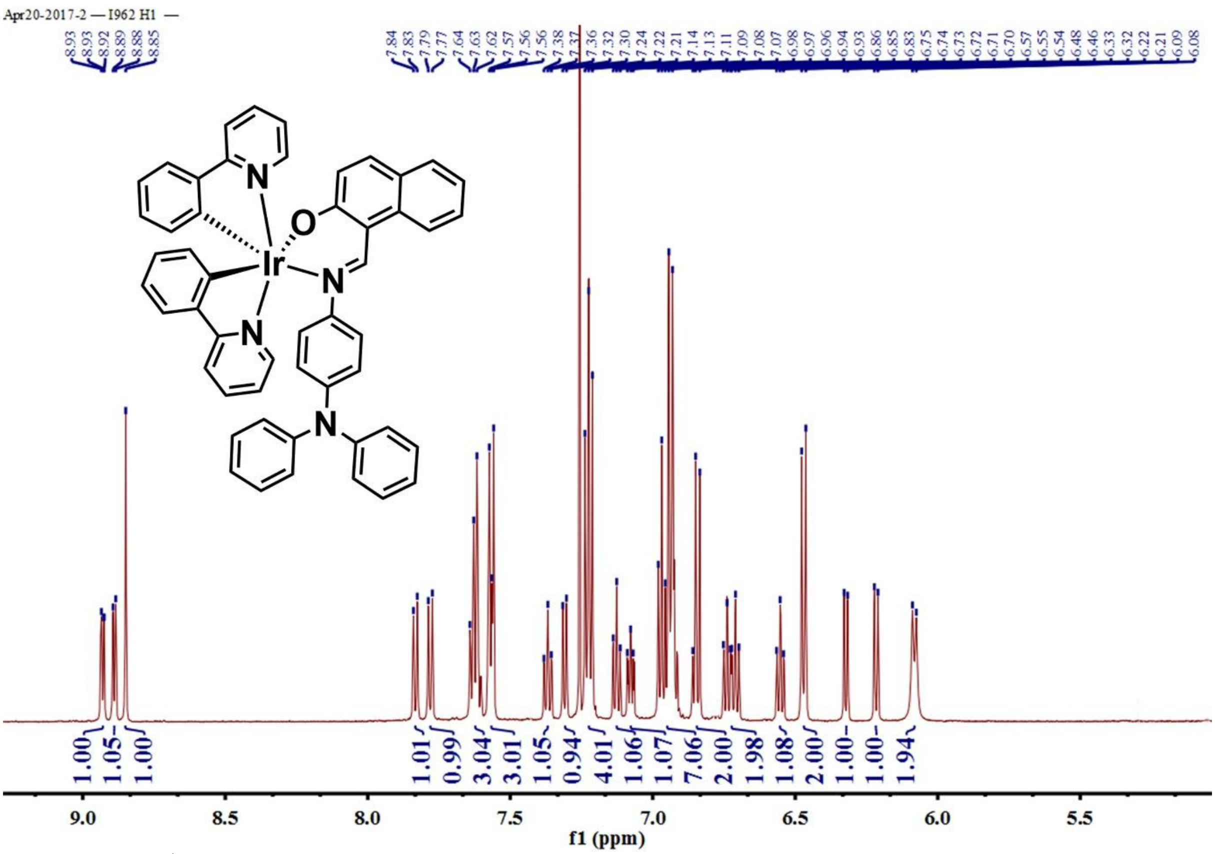

Figure S10. ^{1}H NMR spectrum of **PS1** in $CDCl_3$.

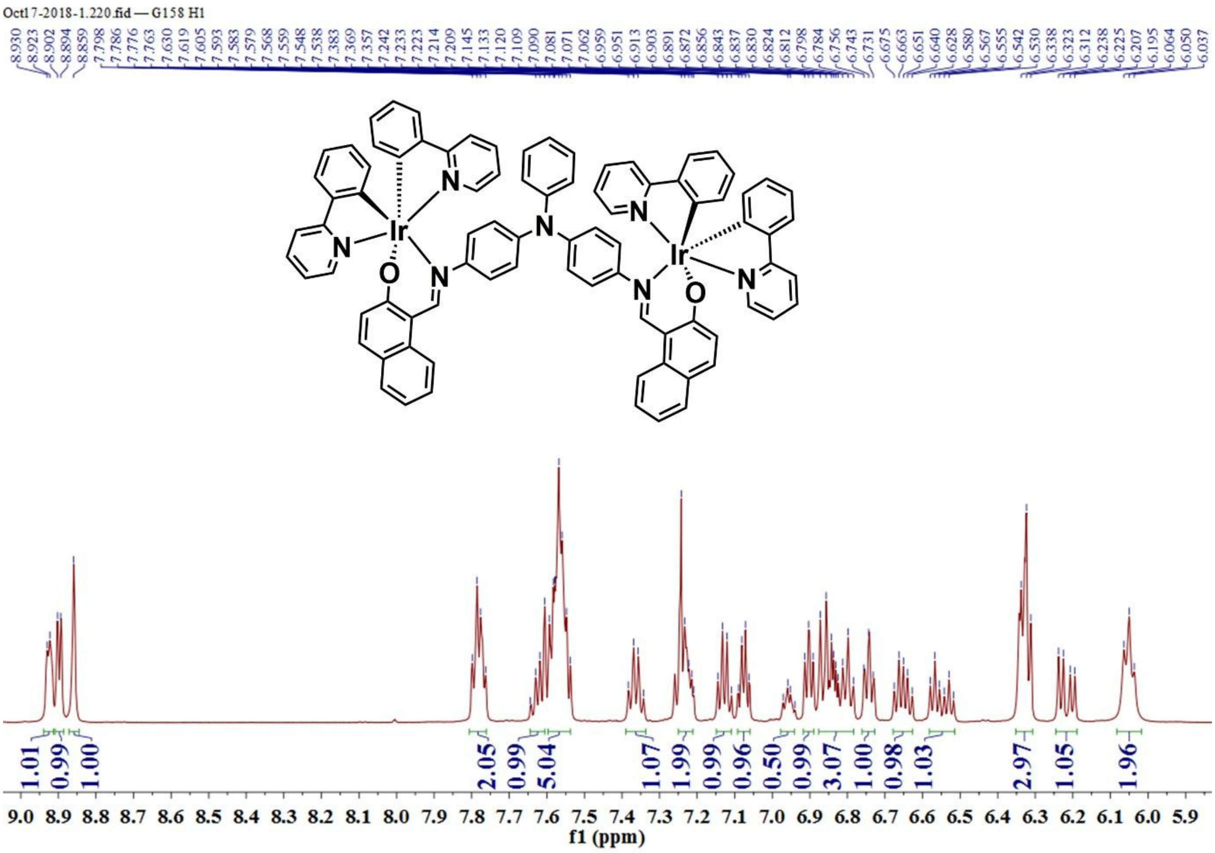

Figure S11. ^{1}H NMR spectrum of **PS2** in $CDCl_3$.

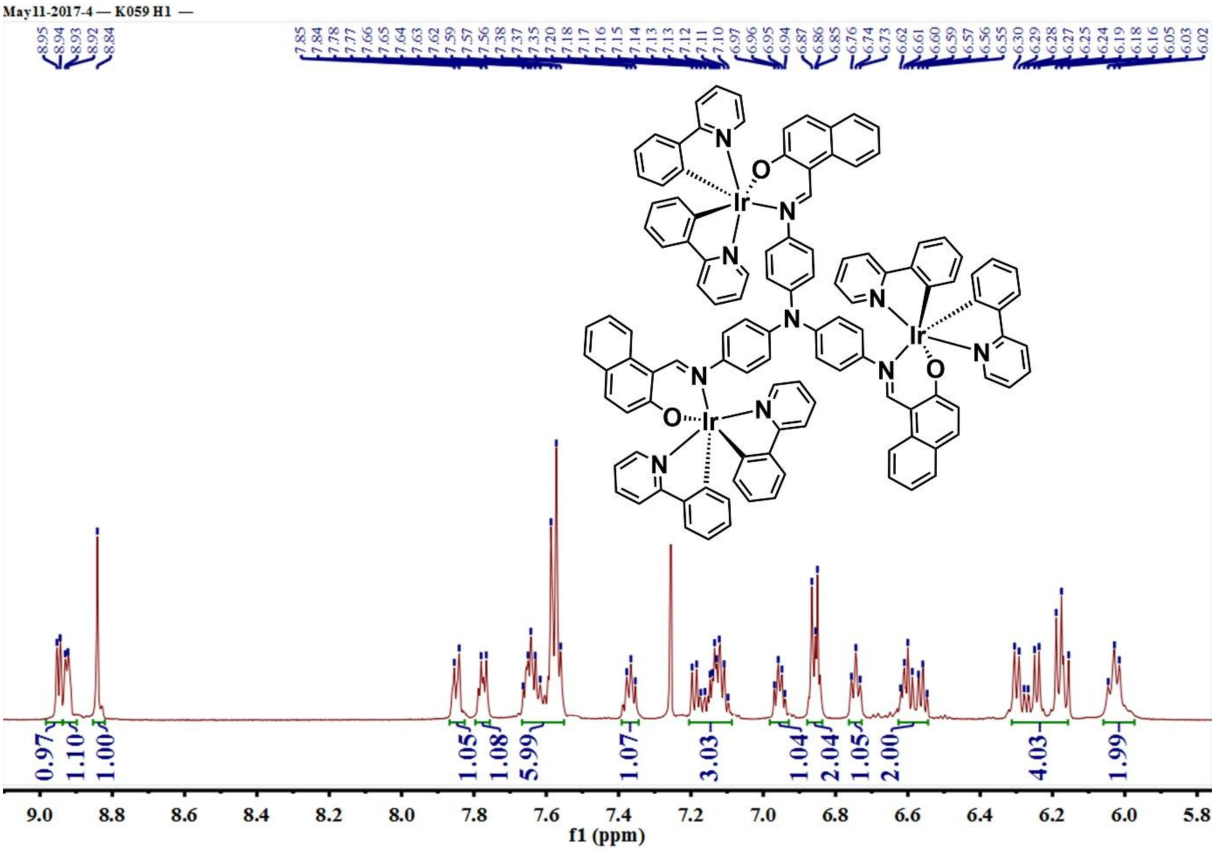

Figure S12. ^{1}H NMR spectrum of **PS3** in $CDCl_3$.

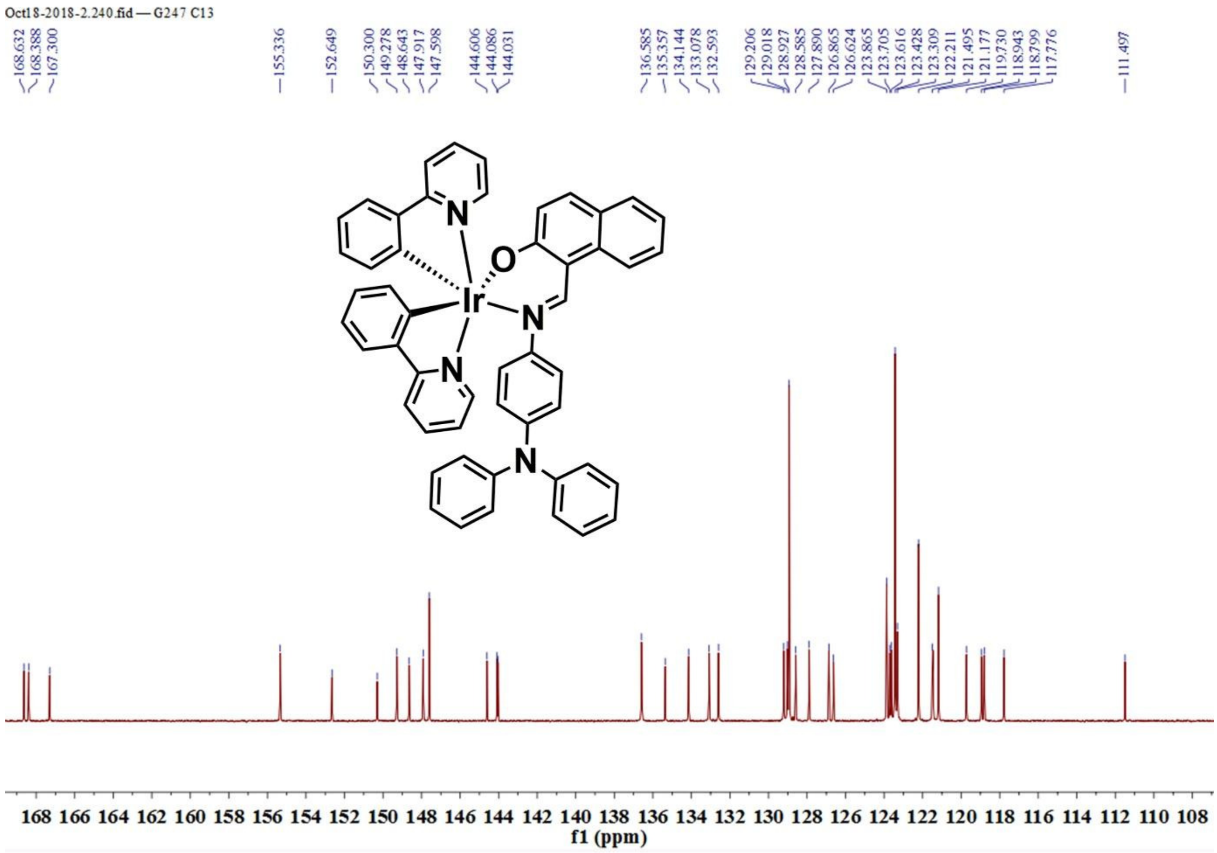

Figure S13. ^{13}C NMR spectrum of **PS1** in $CDCl_3$.

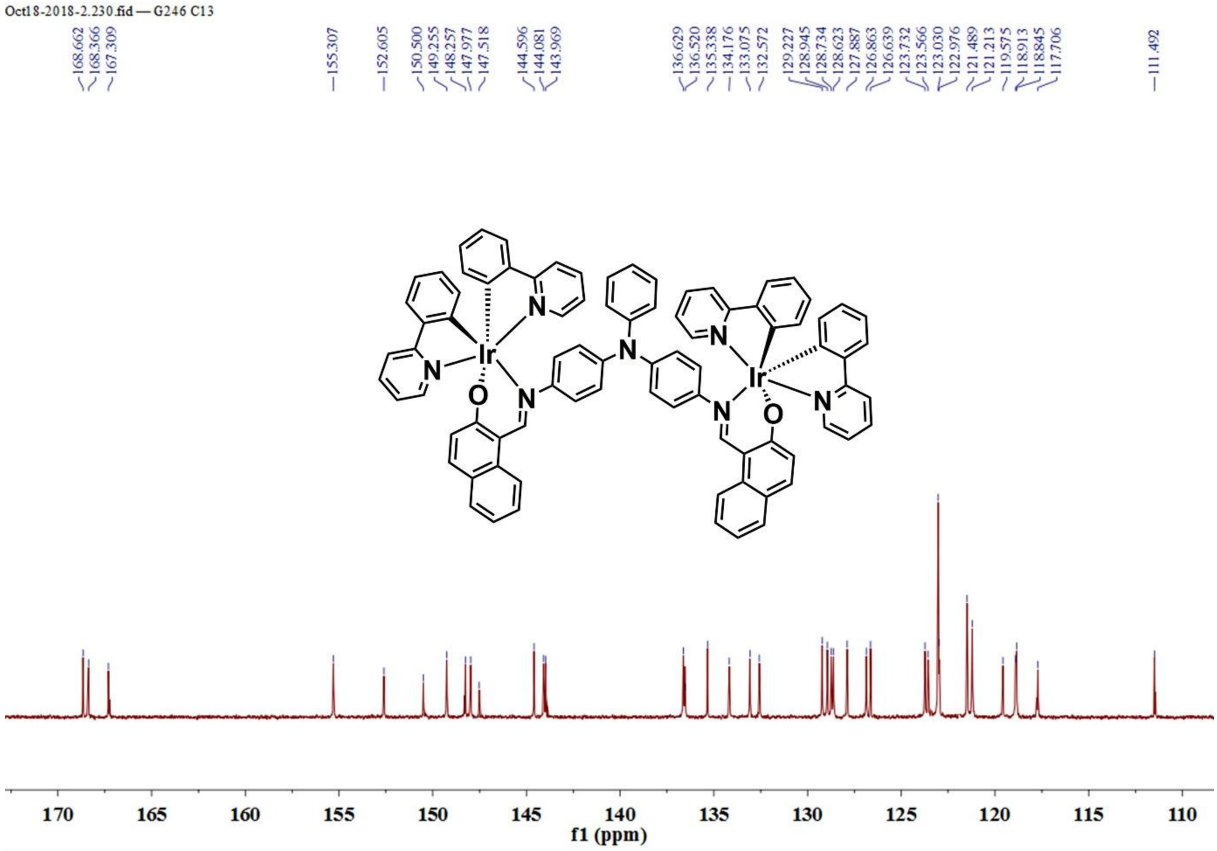

Figure S14. ^{13}C NMR spectrum of **PS2** in $CDCl_3$.

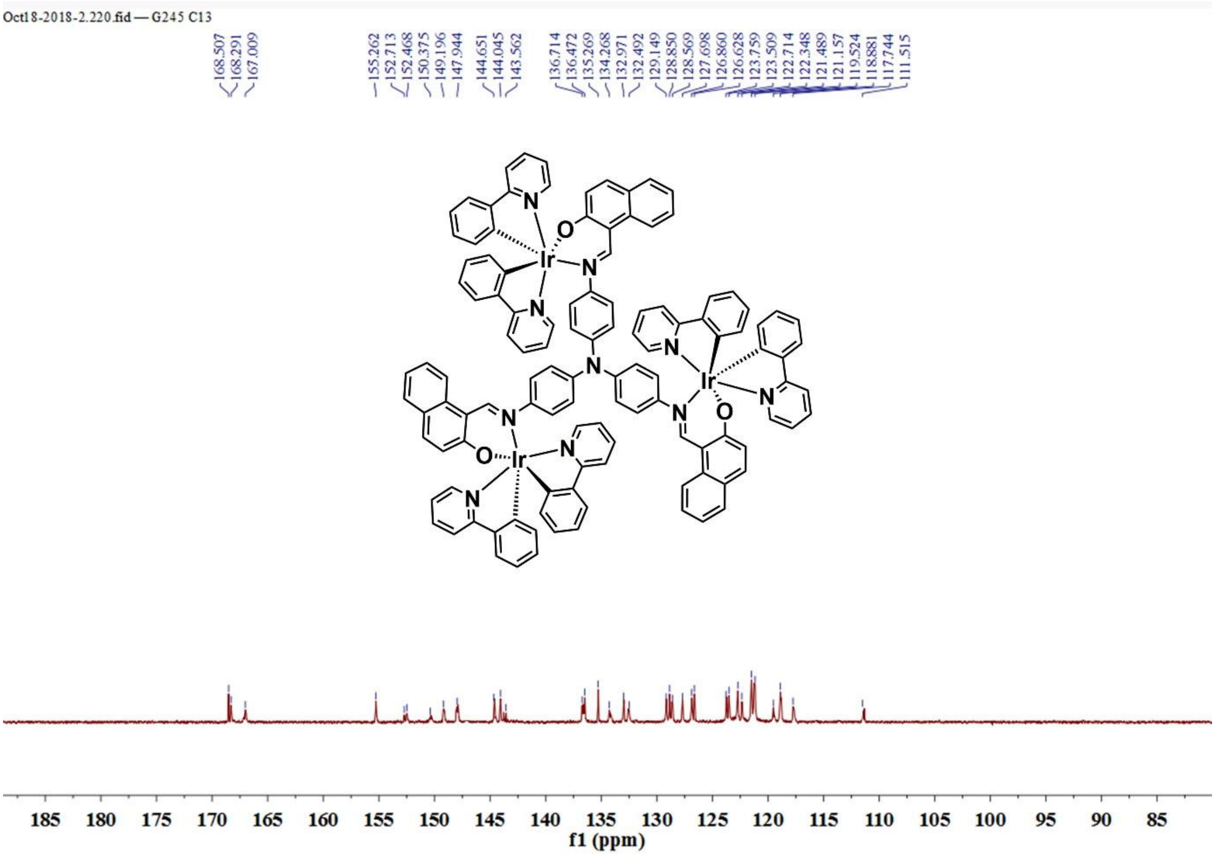

Figure S15. ^{13}C NMR spectrum of **PS3** in $CDCl_3$.

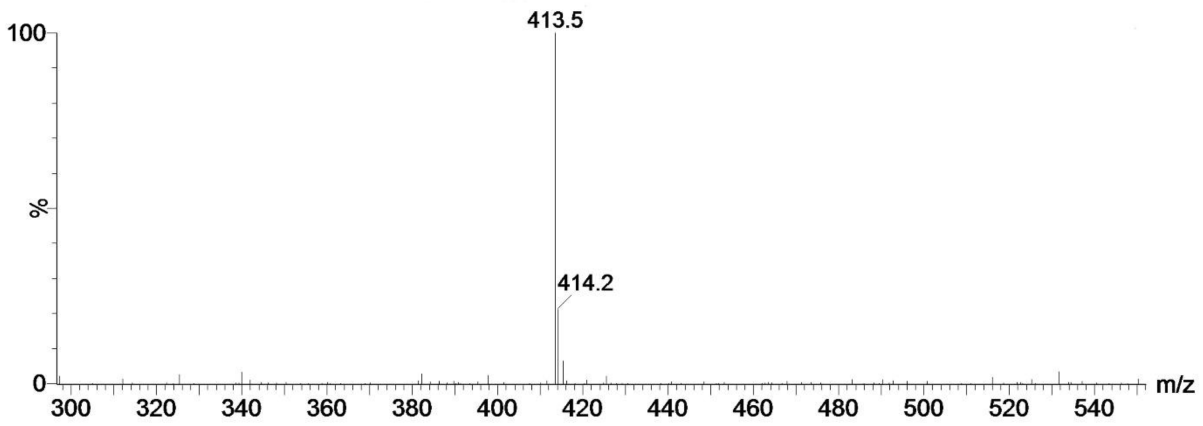

Figure S16. ESI mass spectrum of **L1**.

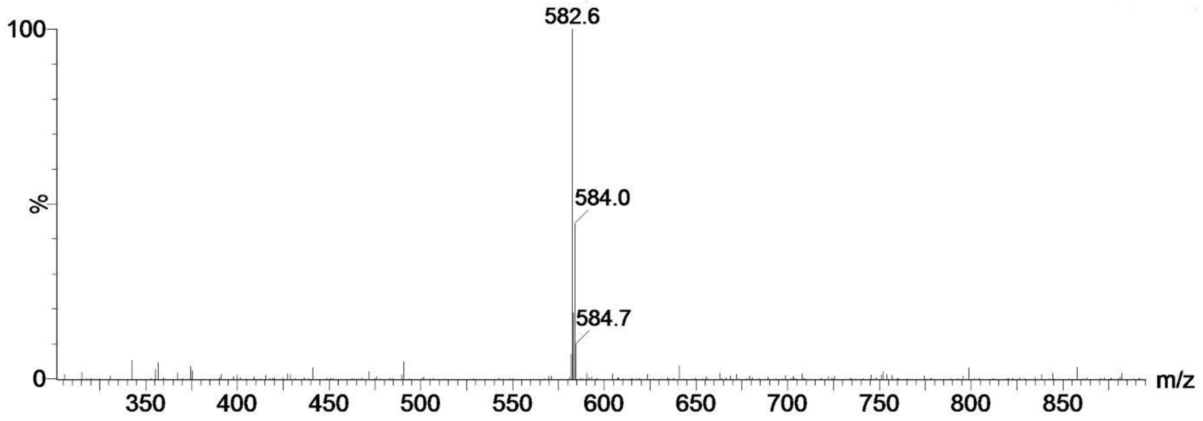

Figure S17. ESI-TOF mass spectrum of **L2**.

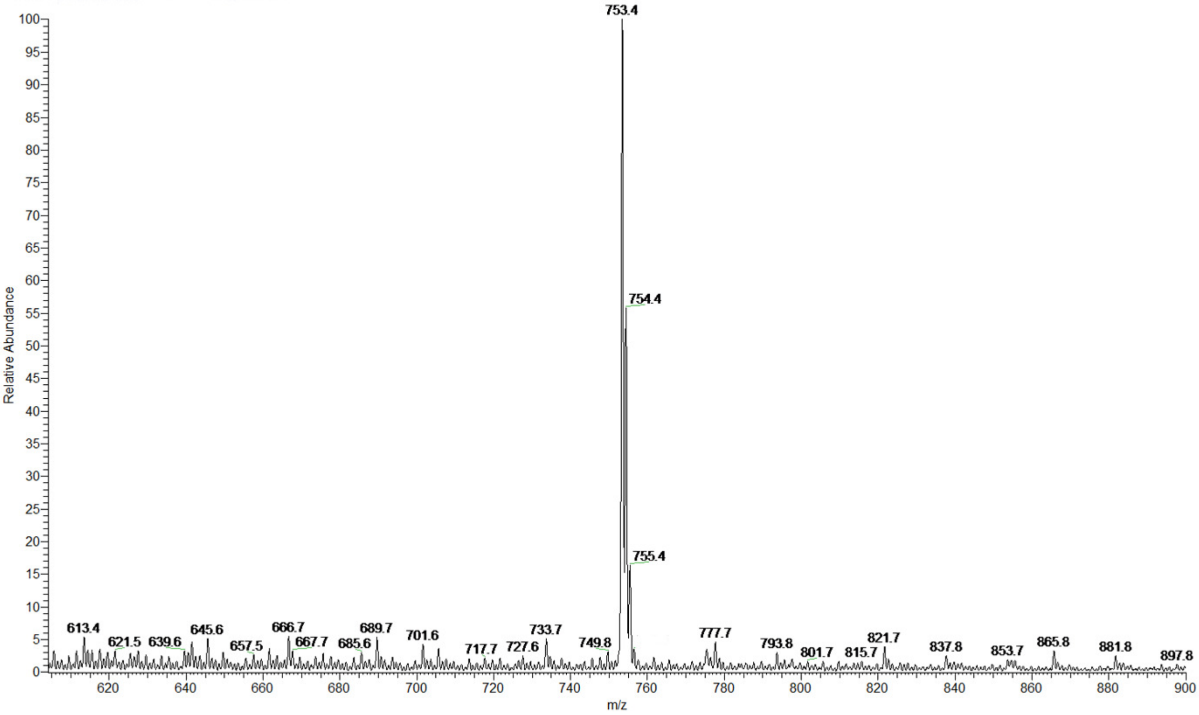

Figure S18. ESI-TOF mass spectrum of **L3**.

.

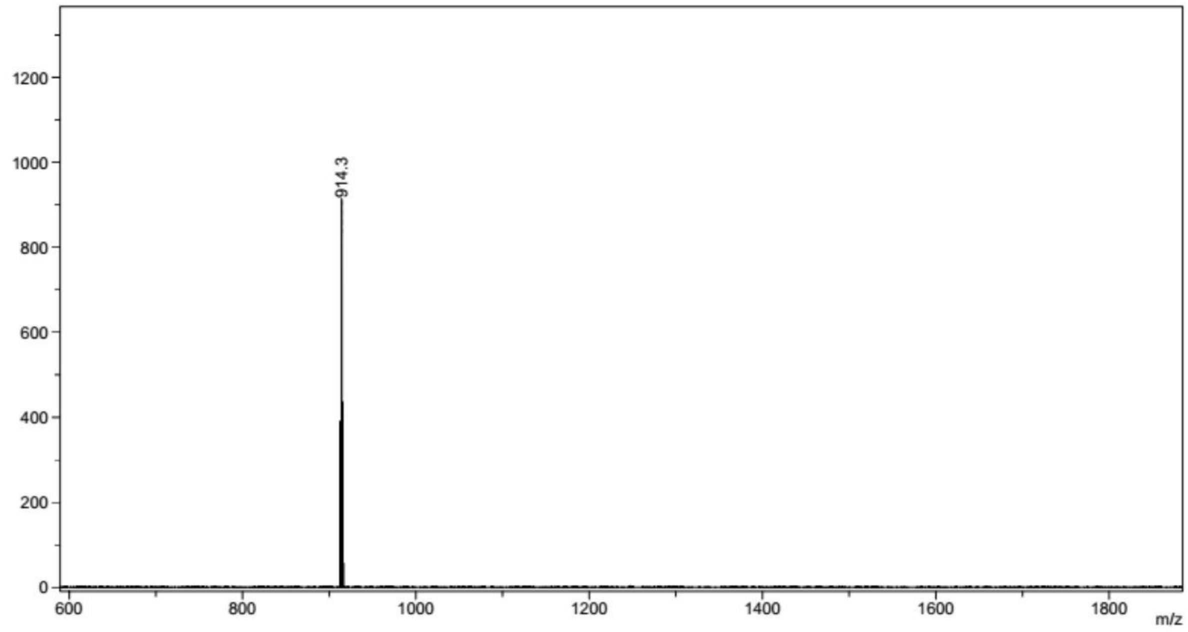

Figure S19. MALDI-TOF mass spectrum of **PS1**.

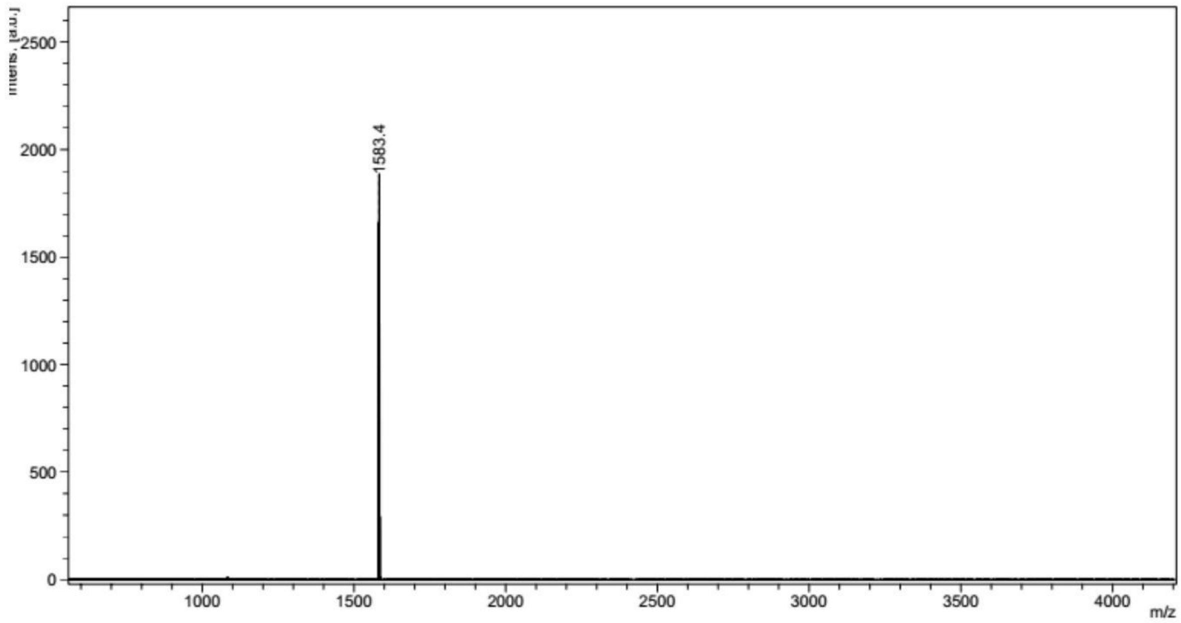

Figure S20. MALDI-TOF mass spectrum of **PS2**.

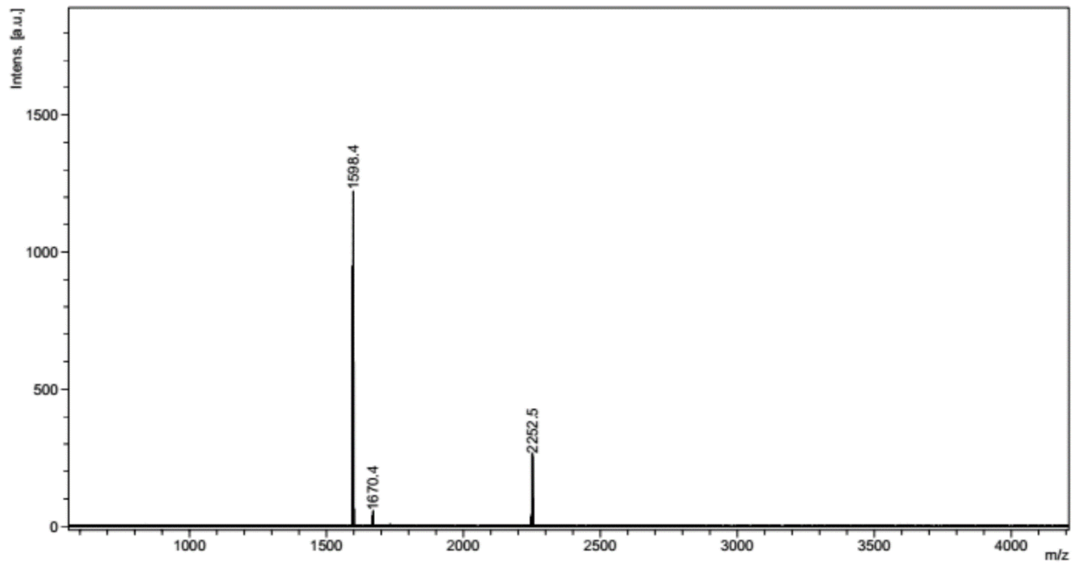

Figure S21. MALDI-TOF mass spectrum of **PS3**.

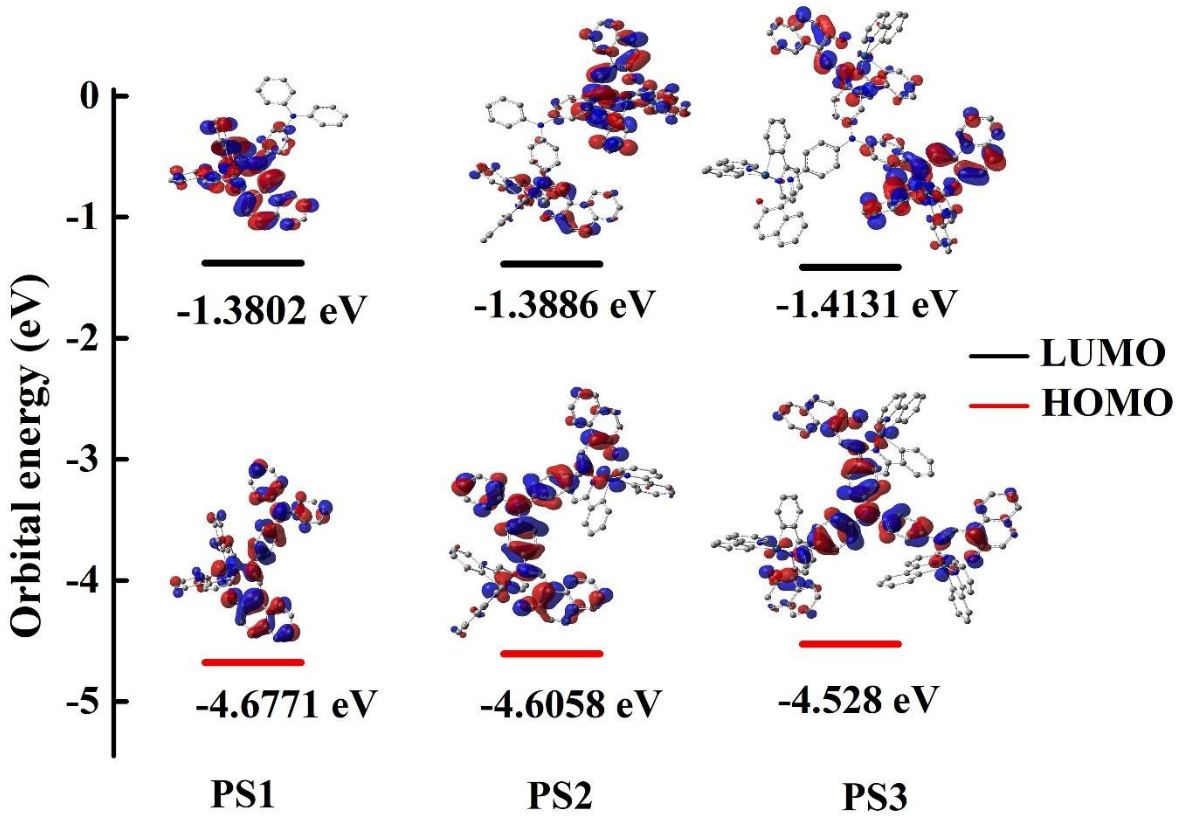

Figure S22. Molecular orbital diagrams, HOMO and LUMO energies for **PS1**, **PS2** and **PS3**.

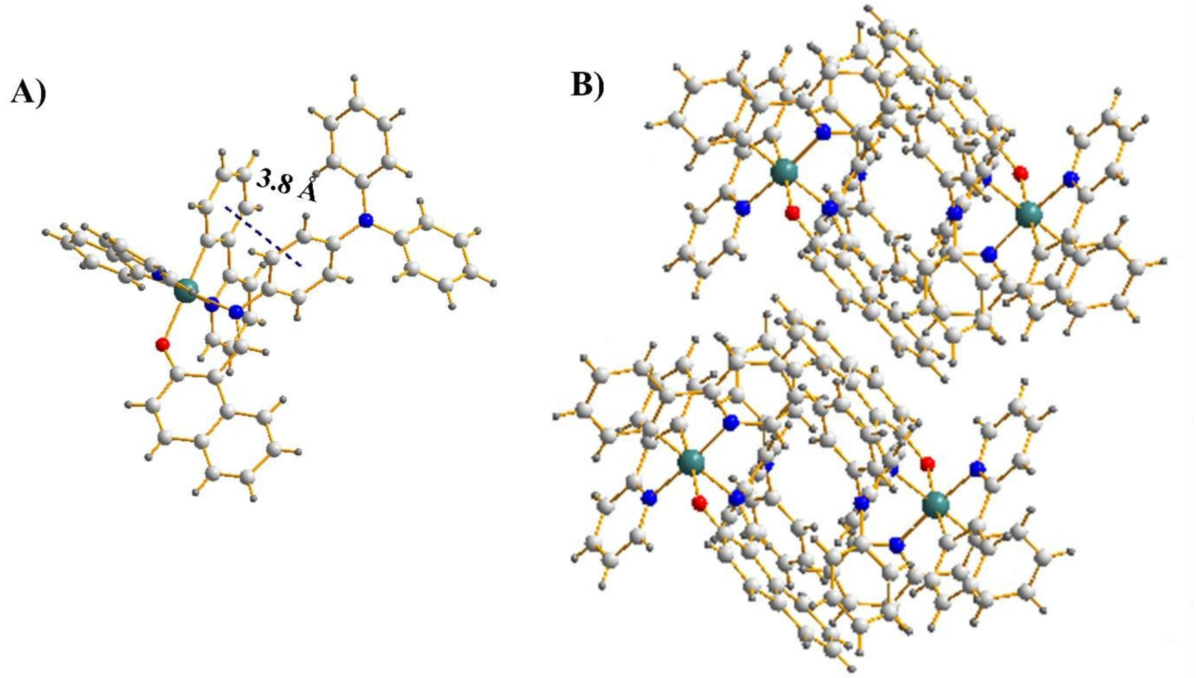

Figure S23. A) The X-ray molecular structure of **PS1** showing intramolecular π–π interactions (dashed line); **B)** Molecular packing of **PS1** in the crystal. Color code: Ir green; N blue; O red. The data have been deposited at the Cambridge Crystallography Data Centre: CCDC number 1866977.

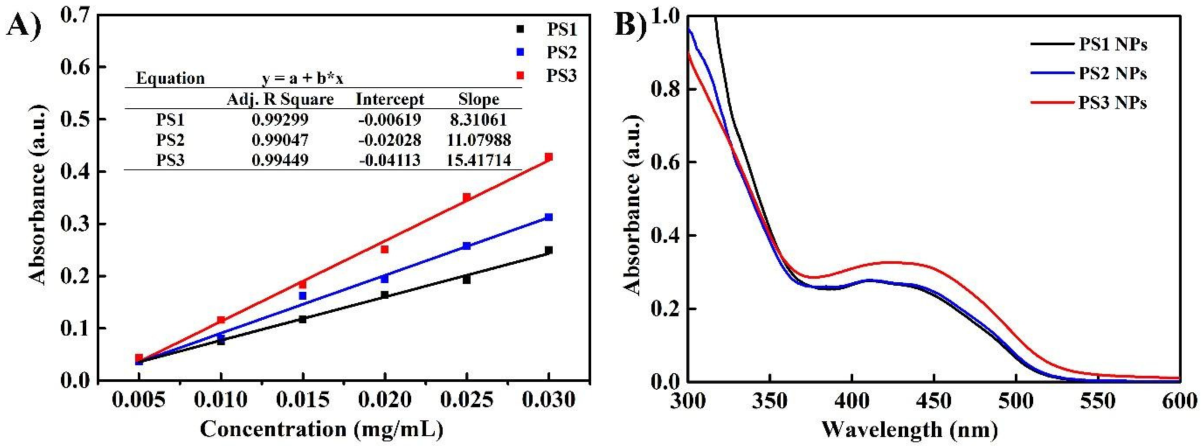

Figure S24. A) Standard curve of **PS1**, **PS2** and **PS3** in THF and water (4:1 v/v); B) the UV-visible absorption spectrum of **PS1** NPs, **PS2** NPs and **PS3** NPs (1 mL) in THF (4mL).

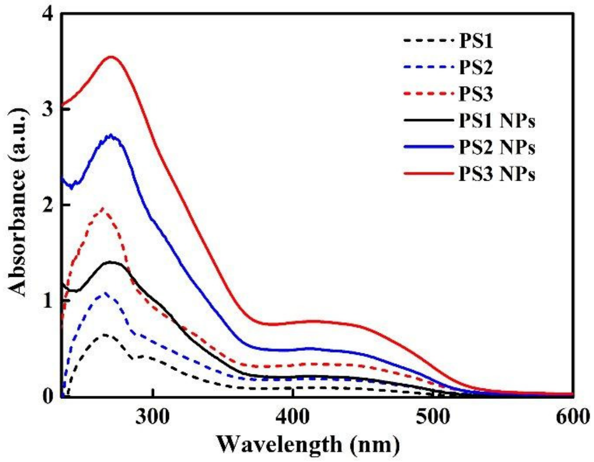

Figure S25. UV-vis absorption spectrum of **PS1**, **PS2** and **PS3** (10^{-5} M) in THF, **PS1** NPs, **PS2** NPs and **PS3** NPs (10^{-5} M) in water.

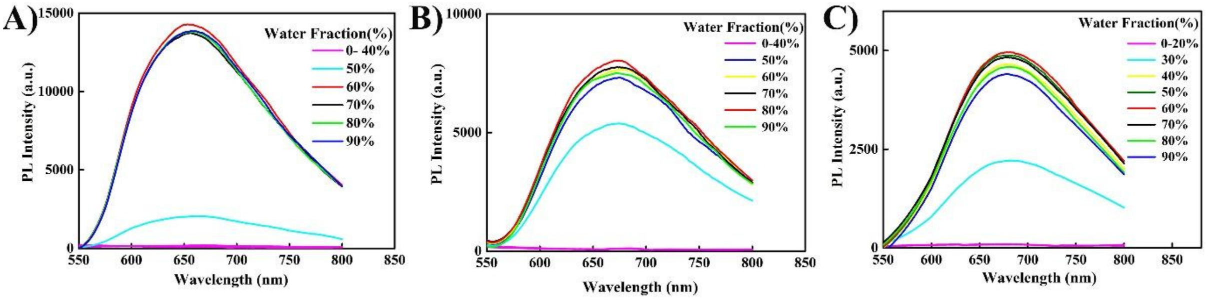

Figure S26. Emission spectra of A) **PS1**, B) **PS2** and C) **PS3** (10^{-5} M) in THF–water mixtures with different water fractions (0–90% v/v) at room temperature.

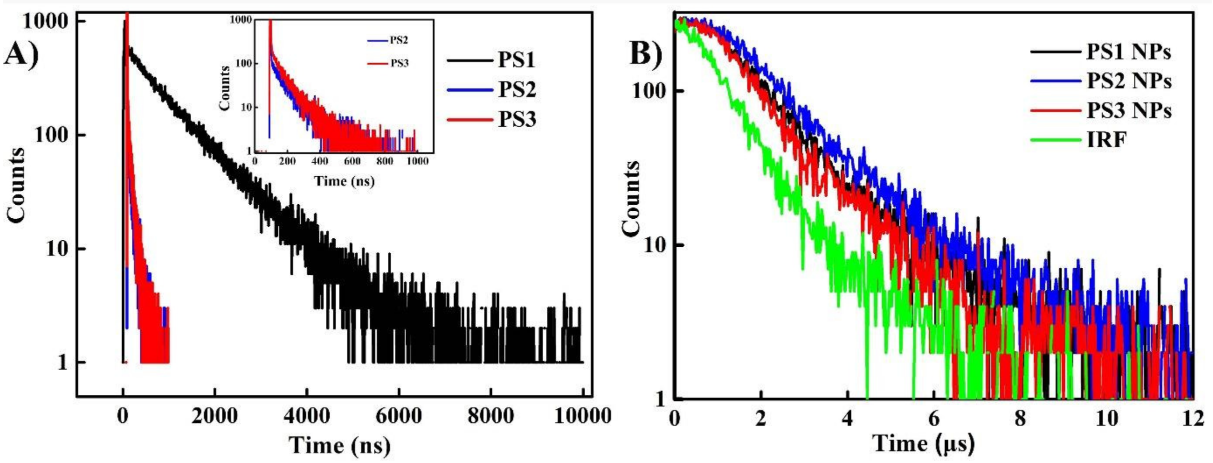

Figure S27. A) Phosphorescence lifetimes of **PS1**, **PS2** and **PS3**. Inset: phosphorescence lifetimes of **PS2** and **PS3**; B) Phosphorescence lifetimes of **PS1** NPs, **PS2** NPs and **PS3** NPs.

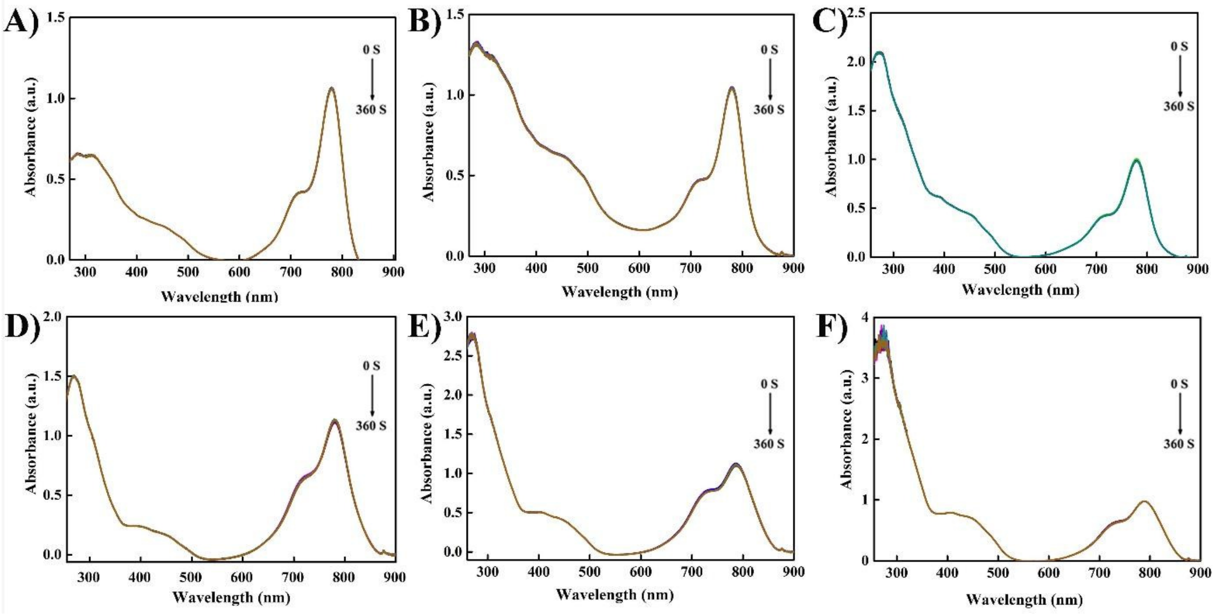

Figure S28. UV–vis absorption spectra changes of ICG (6.5×10^{-6} M, 790 nm) in the presence of A) **PS1**, B) **PS2**, C) **PS3**, D) **PS1** NPs, E) **PS2** NPs and F) **PS3** NPs (10^{-5} M) for different times.

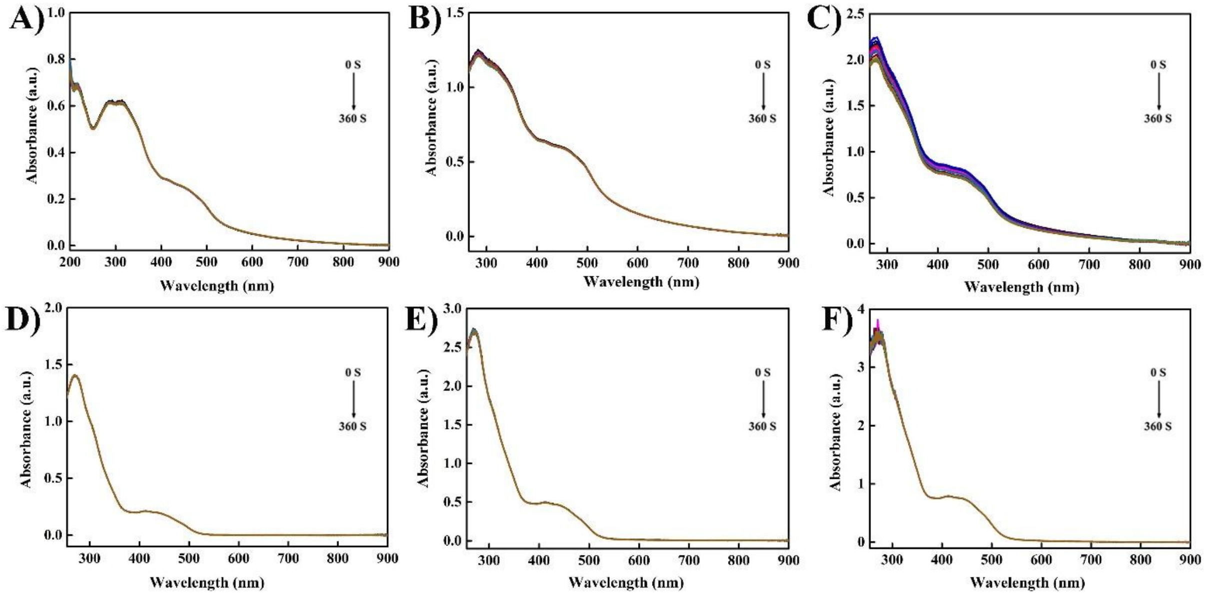

Figure S29. UV–vis absorption spectra changes of A) **PS1**, B) **PS2**, C) **PS3**, D) **PS1** NPs, E) **PS2** NPs and F) **PS3** NPs (10^{-5} M) for different times under irradiation (450 nm, 20 mW cm^{-2}).

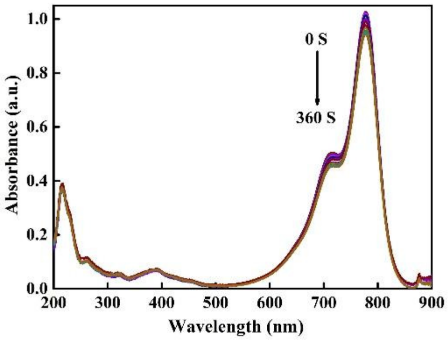

Figure S30. UV–vis absorption spectra of ICG (6.5×10^{-6} M, 790 nm) for different times under irradiation (450 nm, 20 mW cm^{-2}).

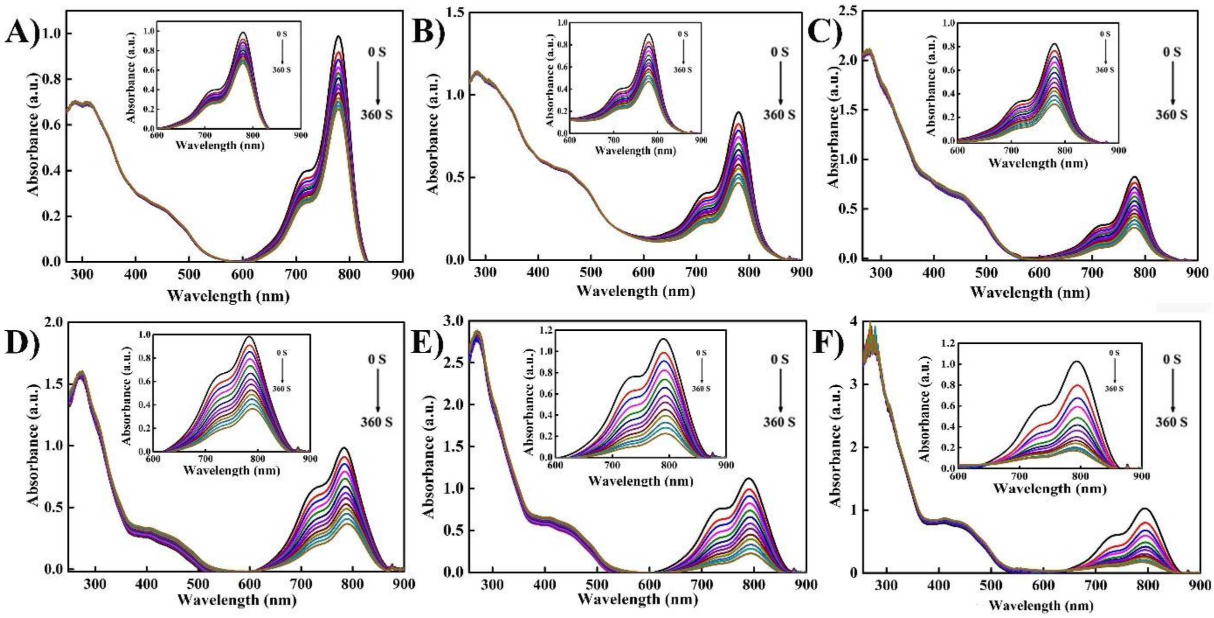

Figure S31. UV–vis absorption spectra of ICG (6.5×10^{-6} M, 790 nm) in the presence of A) **PS1**, B) **PS2**, C) **PS3**, D) **PS1** NPs, E) **PS2** NPs and F) **PS3** NPs (10^{-5} M) for different times under irradiation (450 nm, 20 mW cm^{-2}).

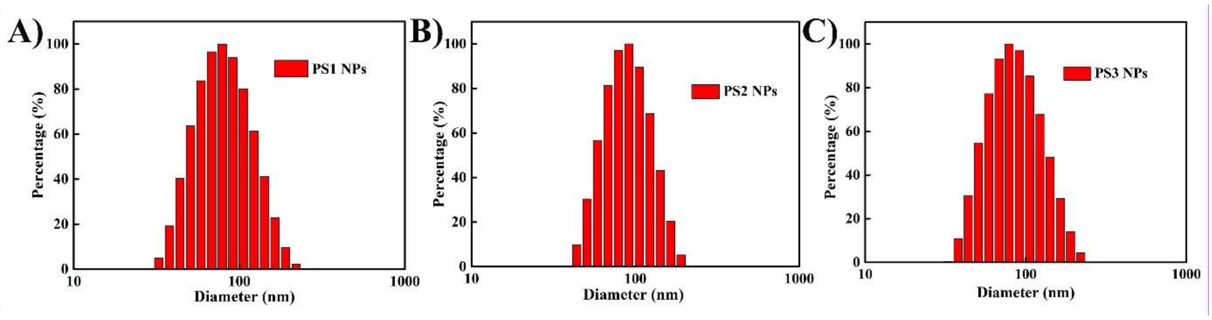

Figure S32. Dynamic laser scattering results of A) **PS1** NPs, B) **PS2** NPs and C) **PS3** NPs in water.

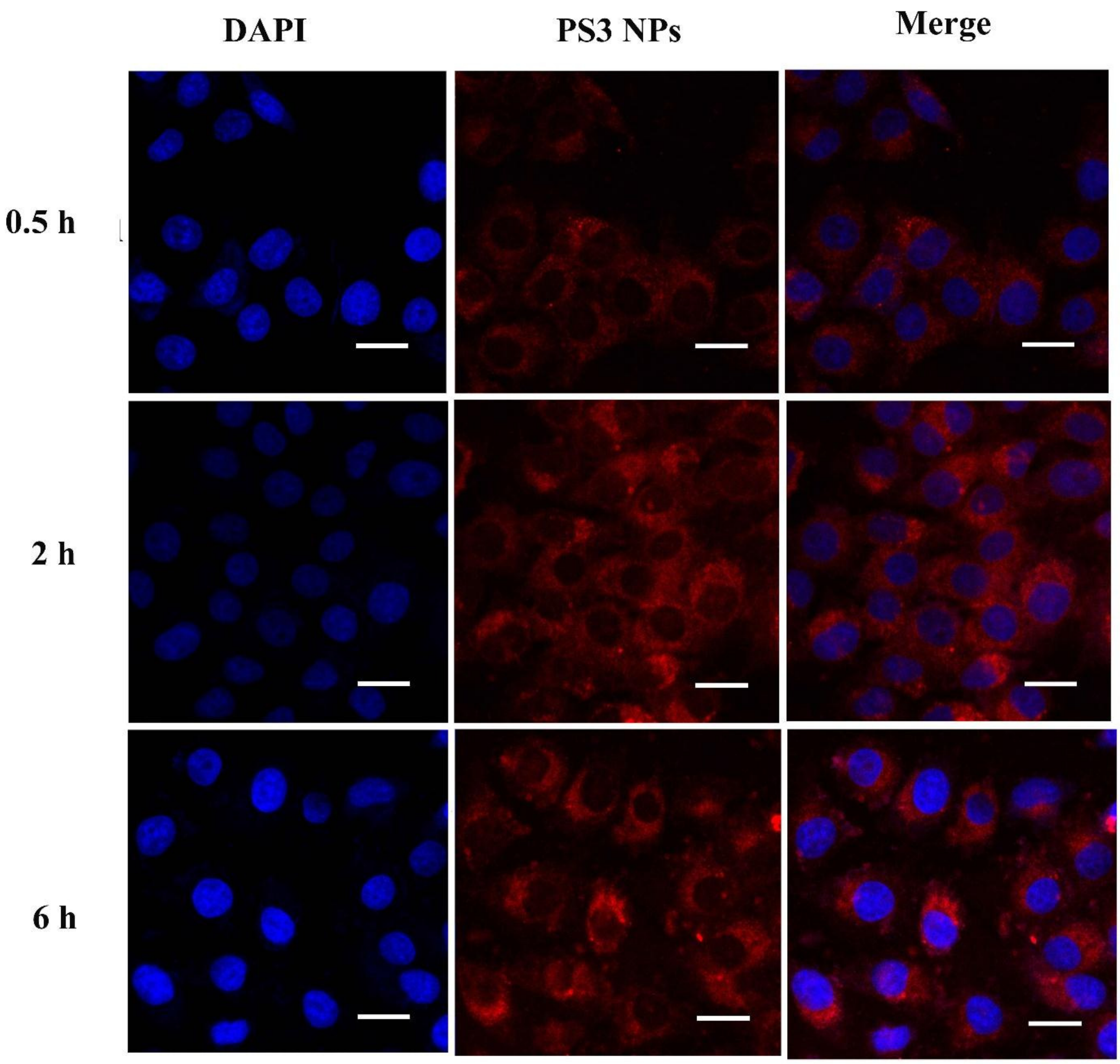

Figure S33. CLSM images of HeLa cells incubated with **PS3** NPs (20 μg mL^{-1}) for 0.5 h, 2 h and 6 h. The scale bars are 20 μm.

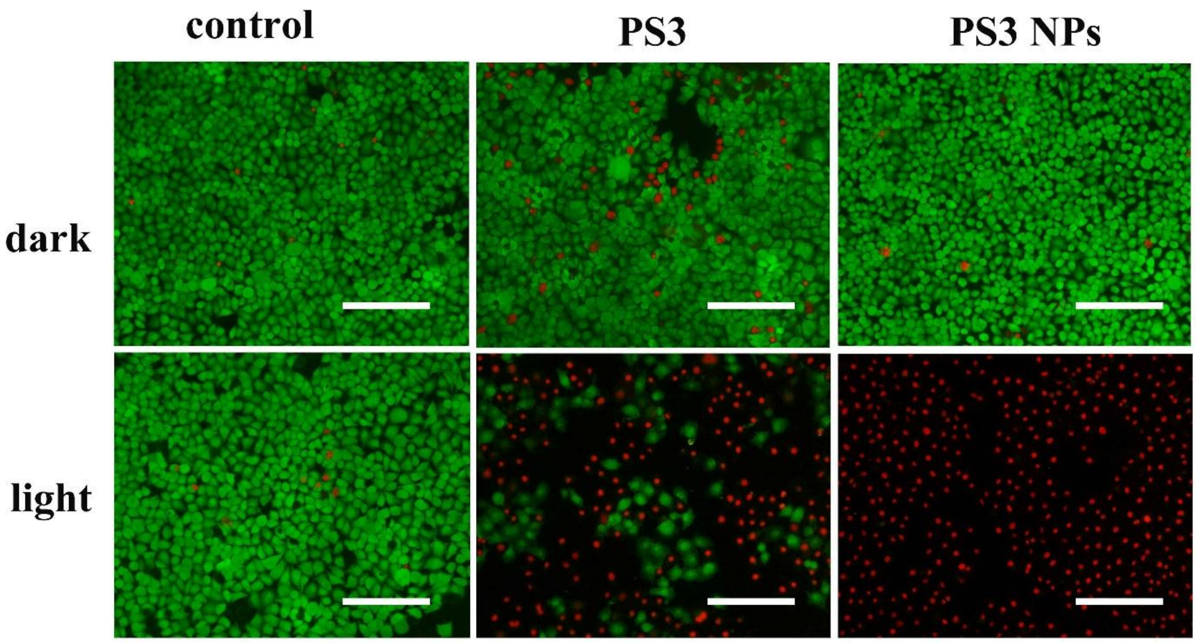

Figure S34. Live/dead cell of **PS3** and **PS3** NPs (10 μg mL^{-1}) staining was tested by using calcein-AM (green fluorescence for live cells) and propidium iodide (PI, red fluorescence for dead cells). The scale bars are 40 μm.

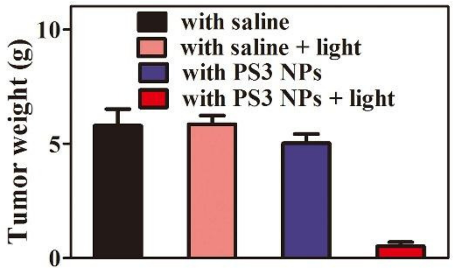

Figure S35. Tumor weights of four groups.

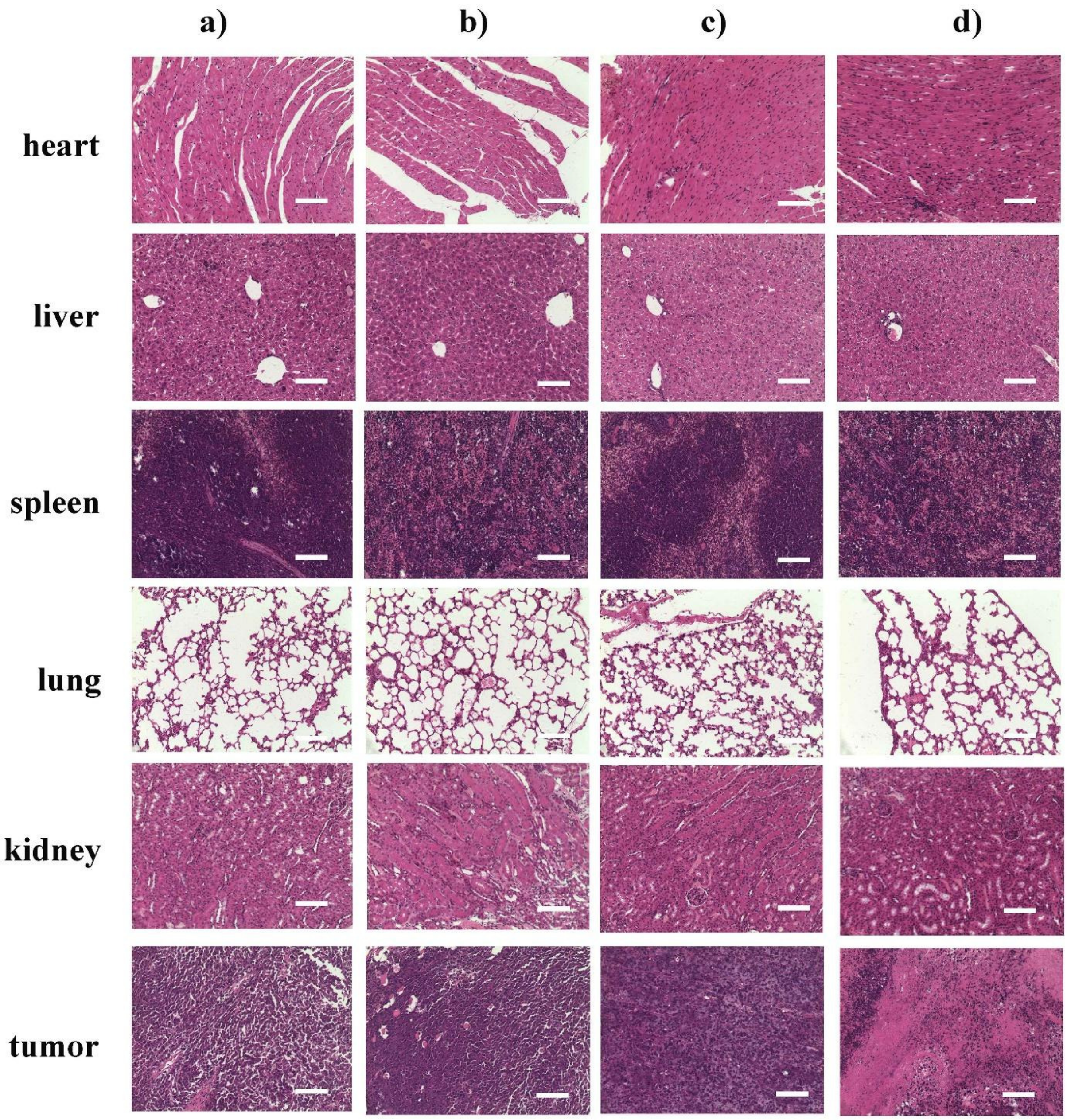

Figure S36. H&E staining of various organs from mice at the end of experiments after treatment a) with saline, b) with saline and light, c) with **PS3** NPs, d) with **PS3** NPs and light.

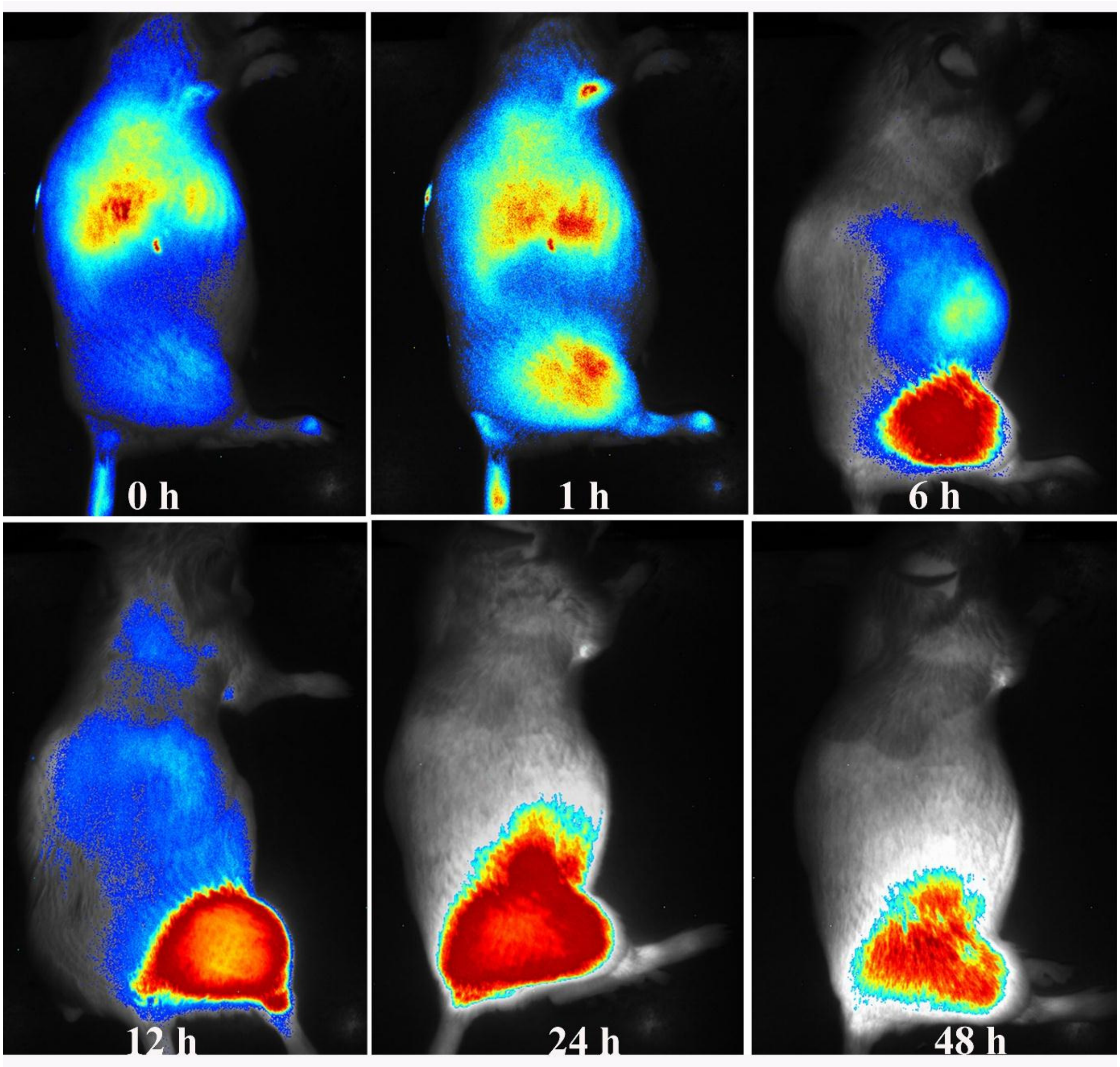

Figure S37. Time-dependent in vivo fluorescence images of H22 tumor-bearing mice after intravenous injection with IR780-labeled **PS3** NPs (100 μg mL^{-1}, 200 μL).

Table S1 Photophysical data of **PS1**, **PS2**, **PS3** and the corresponding NPs.

	λ_{abs} (nm)	λ_{em} (nm)	Φ_p (%)	τ_p (ns)	k_r [d] ($\times10^5$ s^{-1})	k_{nr} [d] ($\times10^6$ s^{-1})
**PS1** [a]	275；413	652	11	959.17	1.14	9.27
**PS2** [b]	271；415	671	5	90.32	5.53	10.51
**PS3** [a]	271；414	690	8	75.98	10.53	12.11
**PS1** NPs [c]	270；413	652	33	4920	0.67	0.16
**PS2** NPs [c]	269；413	671	15	5380	0.28	0.16
**PS3** NPs [c]	270；410	690	35	4610	0.76	0.14

[a] Measured in THF/water (v/v = 2:3) at 298 K (1.0×10^{-5} M, λ_{ex} = 469 nm); [b] Measured in THF/water (v/v = 1:4) at 298 K (1.0×10^{-5} M, λ_{ex} = 469 nm); [c] Measured in water at 298 K (1.0×10^{-5} M, λ_{ex} = 469 nm); [d] The radiative k_r and non-radiative k_{nr} values in neat film were calculated according to the equations: $k_r = \Phi/\tau$ and $k_{nr} = (1- \Phi)/\tau$, from the quantum yields Φ and the lifetime τ values.

Table S2 The average diameter and polydispersity index (PDI) results of **PS1/ PS2/ PS3** NPs measured by DLS.

Sample	**PS1** NPs	**PS2** NPs	**PS3** NPs
Average diameter (nm)	73.39	83.00	79.57
PDI	0.144	0.126	0.156

Table S3 The drug loading content (DLC) and drug loading efficiency (DLE) results of **PS1/ PS2/ PS3** NPs.[a]

Sample	**PS1** NPs	**PS2** NPs	**PS3** NPs
DLC (mg/mL)	0.14631	0.121517	0.11411
DLE (%)	98.03	82.63	79.88

[a] The unloaded PSs were eliminated by filtration through a 0.2 μm syringe-driven filter

References for the SI

[1] M. J. Frisch, G. W. Trucks, H. B. Schlegel, G. E. Scuseria, M. A. Robb, J. R. Cheeseman, G. Scalmani, V. Barone, B. Mennucci, G. A. Petersson, H.Nakatsuji, M. Caricato, X. Li, H. P. Hratchian, A. F. Izmaylov, J. Bloino, G. Zheng, J. L. Sonnenberg, M. Hada, M. Ehara, K. Toyota, R. Fukuda, J. Hasegawa, M. Ishida, T. Nakajima, Y. Honda, O. Kitao, H. Nakai, T. Vreven, J. A. Montgomery, Jr., J. E. Peralta, F. Ogliaro, M. Bearpark, J. J. Heyd, E. Brothers, K. N. Kudin, V. N. Staroverov, R. Kobayashi, J. Normand, K. Raghavachari, A. Rendell, J. C. Burant, S. S. Iyengar, J. Tomasi, M. Cossi, N. Rega, J. M. Millam, M. Klene, J. E. Knox, J. B. Cross, V. Bakken, C. Adamo, J. Jaramillo, R. Gomperts, R. E. Stratmann, O. Yazyev, A. J. Austin, R. Cammi, C. Pomelli, J. W. Ochterski, R. L. Martin, K. Morokuma, V. G. Zakrzewski, G. A. Voth, P. Salvador, J. J. Dannenberg, S. Dapprich, A.

D. Daniels, Ö. Farkas, J. B. Foresman, J. V. Ortiz, J. Cioslowski, D. J. Fox, Gaussian09 (RevisionD.01), Gaussian, Inc., Wallingford, Connecticut, 2009.
[2] K. A. King, P. J. Spellane, R. J. Watts, J. Am. Chem. Soc. **1985**, 107, 1431.
